# Emergence of SGLT2 Inhibitors as Powerful Antioxidants in Human Diseases

**DOI:** 10.3390/antiox10081166

**Published:** 2021-07-22

**Authors:** Kai-Fan Tsai, Yung-Lung Chen, Terry Ting-Yu Chiou, Tian-Huei Chu, Lung-Chih Li, Hwee-Yeong Ng, Wen-Chin Lee, Chien-Te Lee

**Affiliations:** 1 Division of Nephrology, Department of Internal Medicine, Kaohsiung Chang Gung Memorial Hospital and Chang Gung University College of Medicine, Kaohsiung 83301, Taiwan; b9302095@cgmh.org.tw (K.-F.T.); cyang@cgmh.org.tw (T.T.-Y.C.); r5239@cgmh.org.tw (L.-C.L.); stan@cgmh.org.tw (H.-Y.N.); 2 Section of Cardiology, Department of Internal Medicine, Kaohsiung Chang Gung Memorial Hospital, Kaohsiung 83301, Taiwan; lung@cgmh.org.tw; 3 Graduate Institute of Clinical Medical Sciences, College of Medicine, Chang Gung University, Taoyuan 33302, Taiwan; 4 School of Medicine, Chung Shan Medical University, Taichung 40201, Taiwan; 5 Department of Medical Research, Kaohsiung Chang Gung Memorial Hospital, Kaohsiung 83301, Taiwan; skbboyz@cgmh.org.tw; 6 Biobank and Tissue Bank, Kaohsiung Chang Gung Memorial Hospital, Kaohsiung 83301, Taiwan; 7 Institute for Translational Research in Biomedicine, Kaohsiung Chang Gung Memorial Hospital, Kaohsiung 83301, Taiwan

**Keywords:** SGLT2 inhibitors, antioxidants, diabetes, cardiovascular diseases, nephropathy, liver diseases, neural disorders, cancers

## Abstract

Sodium-glucose cotransporter 2 (SGLT2) inhibitors are a new class of oral glucose-lowering agents. Apart from their glucose-lowering effects, large clinical trials assessing certain SGLT2 inhibitors have revealed cardiac and renal protective effects in non-diabetic patients. These excellent outcomes motivated scientists and clinical professionals to revisit their underlying mechanisms. In addition to the heart and kidney, redox homeostasis is crucial in several human diseases, including liver diseases, neural disorders, and cancers, with accumulating preclinical studies demonstrating the therapeutic benefits of SGLT2 inhibitors. In the present review, we aimed to update recent advances in the antioxidant roles of SGLT2 inhibitors in common but debilitating human diseases. We anticipate that this review will guide new research directions and novel therapeutic strategies for diabetes, cardiovascular diseases, nephropathies, liver diseases, neural disorders, and cancers in the era of SGLT2 inhibitors.

## 1. Introduction

Sodium-glucose cotransporter 2 (SGLT2) inhibitors are a new class of oral glucose-lowering agents that block renal glucose reabsorption. SGLT2 inhibitors approved by the United States Food and Drug Administration (FDA) and/or similar bureau in the European Union and other countries include empagliflozin, dapagliflozin, canagliflozin, ertugliflozin, ipragliflozin, tofogliflozin, luseogliflozin, and remogliflozin [1]. In addition to lowering blood glucose, SGLT2 inhibitors can reduce body weight, improve visceral adiposity, lower blood pressure, and normalize lipid profile and serum uric acid levels [2,3,4,5]. Notably, recent cardiovascular outcome trials (CVOTs) assessing SGLT2 inhibitors have shown improvements in cardiovascular and renal outcomes in patients with and without type 2 diabetes mellitus (T2DM) [6,7,8,9,10,11,12]. Based on accumulating evidence, the American Diabetes Association (ADA)/European Association of the Study for Diabetes (EASD) recommends SGLT2 inhibitors as the mainstay of treatment for T2DM [13].

### 1.1. Potential Antioxidant Roles of SGLT2 Inhibitors in Cardiorental Benefits in Landmark Clinical Trials

Several mechanisms underlying the renal and cardiovascular benefits of SGLT2 inhibitors have been proposed. SGLT2 inhibitors have been shown to directly reduce high glucose-induced oxidative stress in the proximal tubules [14,15,16]. Other mechanisms include reduced glomerular hyperfiltration via tubuloglomerular feedback, reduced inflammation, ameliorated fibrosis, attenuated sympathetic nervous system activation, and improved mitochondrial function [14,17,18,19]. Interestingly, SGLT2 is not expressed in the adult heart. Proposed mechanisms explaining the cardioprotective effects of SGLT2 inhibitors include osmotic diuresis, natriuresis, improved renal function, blood pressure reduction, improved vascular function, increased hematocrit, changes in tissue sodium handling, reduced adipose tissue-mediated inflammation and proinflammatory cytokine production, a shift toward ketone bodies as the metabolic substrate, lowered serum uric acid levels, suppression of advanced glycation end product (AGE) signaling, and decreased oxidative stress [1,20]. Most of these mechanisms account for the regulatory interplay between the kidney and heart. Notably, SGLT2 inhibitors are capable of reducing oxidative stress in the heart [21] and kidney [14,22]. As potential antioxidants, SGLT2 inhibitors may offer direct cardioprotection and renoprotection.

### 1.2. SGLT2 Inhibitors Reduce Oxidative Stress in Human Diseases

Oxidative stress, defined as an imbalance between pro-oxidants and antioxidants, is a crucial factor underlying the pathogenesis of multiple human diseases including diabetes, cardiovascular diseases, nephropathies, liver diseases, neural disorders, and cancers [23]. SGLT2 inhibitors act as indirect antioxidants due to their ability to reduce high glucose-induced oxidative stress. In addition, SGLT2 inhibitors have been shown to reduce free radical generation [24], suppress pro-oxidants (e.g., NADPH oxidase 4 [NOX4] and thiobarbituric acid-reactive substances [TBARS]) [25,26], and upregulate antioxidant systems such as superoxide dismutases (SODs) and glutathione (GSH) peroxidases [27,28,29]. SGLT2 inhibitors have been shown to suppress cellular proliferation by reducing oxidative stress in multiple types of cancer [30,31,32,33,34]. Detailed discussion addressing specific markers of pro-oxidants and antioxidants that are examined in various studies is presented in subsequent sections under different disease headings.

### 1.3. The Anti-Inflammatory Features of SGLT2 Inhibitors

Inflammation plays important roles in diabetes, chronic kidney disease, cardiovascular disease, liver diseases, neural disorders, and cancers. In various experimental disease models, SGLT2 inhibitors have been demonstrated to exert anti-inflammatory effects [35]. They are able to directly downregulate the expression of proinflammatory mediators including monocyte chemoattractant proteinrac (MCP-1), TGF-β, TNF-α, interleukin-6 (IL-6), nuclear factor κB (NF-κB) and C-reactive protein (CRP) [26,36,37]. In addition to these direct influences of SGLT2 inhibitors on inflammation, SGLT2 inhibitors are also able to attenuate inflammation by regulating renin-angiotensin system (RAS) activity, tissue hemodynamic alterations and the imbalanced redox state [38,39]. Oxidative stress activates a variety of transcription factors, including hypoxia-inducible factor (HIF)-1α, p53, NF-κB, peroxisome proliferator-activated receptor (PPAR) γ, and nuclear factor erythroid 2–related factor 2 (Nrf2), which, in turn, lead to the expression of various chemokines and inflammatory cytokines [40,41]. By reducing oxidative stress, in various disease models, SGLT2 inhibitors have been shown to attenuate inflammation by regulating transcription factors of these chemokines and cytokines. Detailed pathways will be discussed in subsequent sections in a disease-specific fashion.

### 1.4. A Unique Perspective of SGLT2 Inhibitors

Oxidative stress plays a crucial role in numerous common but debilitating human diseases. As a result, reducing oxidative stress acts as a promising strategy to offer the unmet needs of existing treatments of these diseases. Accumulating evidence has demonstrated the therapeutic potential of SGLT2 inhibitors in these diseases, but the antioxidant roles of SGLT2 inhibitors in these diseases are yet to be reviewed.

**Figure 1:**
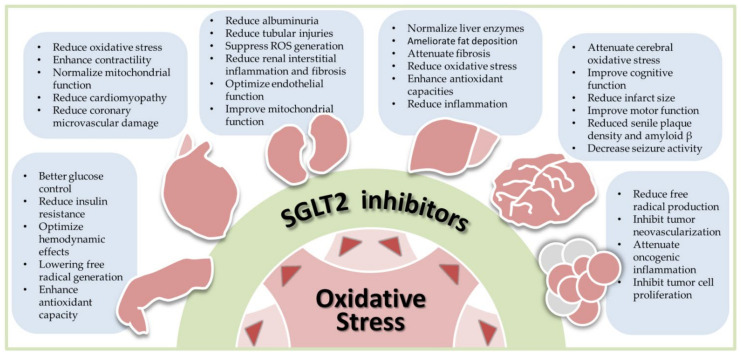
Antioxidant effects of sodium-glucose cotransporter 2 (SGLT2) inhibitors in human diseases, including diabetes, cardiovascular diseases, nephropathy, liver diseases, neural disorders, and cancers. Oxidative stress is commonly seen in human diseases. By reducing oxidative stress, SGLT2 inhibitors exhibit the therapeutic potential in these diseases. The bullet points listed in each blue box summarize results of preclinical and clinical studies in each specific disease. ROS, reactive oxygen species.

SGLT2 inhibitors have received great attention from scientists and clinicians. The pros and cons of SGTL2 inhibitors have been extensively reviewed in a plethora of papers [1,2,5,42,43,44]. Assessment on the research design, outcomes and limitations of large landmark trials on SGLT2 inhibitors have been thoroughly discussed [1,13,45,46] and are beyond the scope of this review. Unique to most current review articles on SGLT2 inhibitors, this review focuses on recent advances in SGLT2 inhibitor research from an antioxidant perspective. We searched the Ovid MEDLINE, PubMed, Embase and Cochrane databases for SGLT2 inhibitors, antioxidants, oxidative stress, diabetes, cardiovascular diseases, kidney, liver, neural disease, and cancers published up to 30 April 2021. We will discuss how SGLT2 inhibitors reduce oxidative stress in human diseases. Although experimental and clinical evidence supporting SGLT2 inhibitors as antioxidants are not equally present in each disease discussed, we structured our review in a diseases-specific fashion. By presenting this disease-specific review, we hope scientists and medical professionals will better understand the antioxidant roles of SGLT2 inhibitors in the disease-of-interest and quickly find out the research gaps in their fields. Figure 1 illustrates the scope of this review.

## 2. SGLT2 Inhibitors as Antioxidants in Diabetes

T2DM is a major threat to human health, impacting 9.3% of adults globally in 2019, thus resulting in several microvascular and macrovascular complications, including retinopathy, nephropathy, peripheral arterial disease, and cardiovascular disease [47]. Oxidative stress is considered a major hallmark of the pathogenesis of T2DM and its complications. In patients with T2DM, hyperglycemia can upregulate chronic inflammation and increase reactive oxygen species (ROS) generation, which ultimately causes endothelial dysfunction and vascular complications; conversely, increased oxidative stress and persistent inflammation can aggravate insulin resistance and exacerbate hyperglycemia in T2DM and prediabetes [48,49]. Furthermore, the dysregulation of mitochondrial oxidation and the complex interplay between nicotinamide adenine dinucleotide phosphate (NADPH) oxidases, xanthine oxidase, endothelial nitric oxide synthase (eNOS), and AGEs contribute to the elevated oxidative stress and hence deteriorate systemic complications of T2DM [49,50,51,52]. To prevent complications and defer the progression of T2DM, appropriate treatment of hyperglycemia and inhibition of oxidative stress are crucial. Several strategies have been proposed to modulate oxidative stress and inflammation in T2DM, including dietary intervention and medications such as antihypertensives, statins, antiplatelets, probiotics, and glucose-lowering agents [49,53,54,55]. Among these interventions, SGLT2 inhibitors have emerged as newly recognized antioxidant agents, affording their effects not only via glucose control but also by lowering free radical generation and regulating the antioxidant systems such as SODs and GSH peroxidases [27,28,29]. In the following subsections, we will review the antioxidant mechanisms underlying SGLT2 inhibitors in T2DM, as well as their possible effects on insulin resistance and outcomes of T2DM.

### 2.1. The Antioxidant Mechanisms Underlying SGLT2 Inhibitors in T2DM

Accumulating evidence from experimental and clinical studies supports the antioxidant roles of SGLT2 inhibitors in T2DM, with mechanisms that involve both inhibition of free radical generation and regulation of antioxidant systems via direct modulative properties, as well as indirect influences from glucose-lowering and hemodynamic effects.

Several studies have described the mechanistic view of SGLT2 inhibitors in free radical suppression during T2DM. Steven et al. examined the antioxidative effects of empagliflozin in Zucker diabetic fatty rats, demonstrating that empagliflozin could prevent the development of systemic oxidative stress, AGE-receptor for AGEs (RAGE) signaling pathway, and inflammation by lowering glucose levels, restoring insulin sensitivity, and improving the redox status [24]. Other studies in diabetic mouse models indicated that SGLT2 inhibitors suppressed NADPH oxidase 4 (NOX4), enhanced Nrf2-heme oxygenase-1 (HO-1)-mediated oxidative stress response, regulated the Sestrin2-mediated adenosine monophosphate-activated protein kinase-mammalian target of rapamycin (AMPK-mTOR) signaling pathway, decreased oxidative stress, and therefore delivered renal, β cell mass, and cardiovascular benefits [25,56]. In a study utilizing human umbilical vein endothelial cells, SGLT2 inhibitors activated the phosphatidylinositol 3-kinase (PI3K)/protein kinase B (AKT)/eNOS signaling pathway, decreased free radical damage, and ameliorated endothelial dysfunction [57]. Additionally, the antioxidant effects of SGLT2 inhibitors involving other pro-oxidant enzymes and proinflammatory cytokines, such as monocyte chemoattractant protein-1 (MCP-1), have been described in animal and in vitro studies [22,58]. Shao et al. and Sa-nguanmoo et al. reported that SGLT2 inhibitors attenuated mitochondrial dysfunction via several pathways, including PPARγ coactivator 1α and mitochondrial transcription factor A signaling in diabetic rat models, which also decreased free radical generation [59,60]. Moreover, in vivo and in vitro findings have indicated that the intrarenal hemodynamic effects and inhibition of the RAS by SGLT2 inhibitors can ameliorate oxidative stress. In clinical studies of dapagliflozin and canagliflozin, decreased 8-hydroxy-2-deoxyguanosine (8-OHdG) and oxidized low-density lipoprotein (ox-LDL) levels in patients with T2DM further corroborated the antioxidant effects of SGLT2 inhibitors [39,61].

Antioxidant systems are defense mechanisms against oxidative stress, and mechanistic evidence has indicated the role of SGLT2 inhibitors in antioxidant enhancement. Oshima et al. revealed that empagliflozin increased antioxidant proteins such as SOD2 and catalase in a diabetic rat model [28]. In addition, beneficial effects on manganese/copper/zinc-dependent SODs and GSH peroxidase were observed in SGLT2 inhibitor-treated animal models [29,39]. Improvements in GSH and GSH reductase profiles in leukocytes were documented in a prospective observational study assessing patients with T2DM [62].

Collectively, SGLT2 inhibitors have multifaceted antioxidant effects in T2DM, involving suppressing free radicals and enhancing the antioxidant system. Possible mechanisms include direct alleviation of mitochondrial dysfunction, modulation of pro-oxidant enzymes and proinflammatory cytokines, and upregulation of antioxidant proteins [25,28,29,56,57,58,59,60,62]. Moreover, SGLT2 inhibitors could indirectly ameliorate oxidative stress in T2DM by decreasing AGE-RAGE interactions, with glucose control and regulation of intrarenal hemodynamics and the RAS [24,39,61].

### 2.2. The Effects of SGLT2 Inhibitors on Insulin Resistance

Insulin resistance is a critical feature in the pathogenesis of T2DM, which also aggravates oxidative stress; conversely, oxidative stress exacerbates insulin resistance, accelerating the progression of prediabetes and T2DM [49]. Emerging investigations have indicated that SGLT2 inhibitors might influence insulin signal transduction, improve peripheral insulin sensitivity, and thus alleviate insulin resistance [63]. Combined with the antioxidant mechanisms mentioned above, SGLT2 inhibitors could disrupt the vicious cycle between insulin resistance and oxidative stress and are therefore an excellent therapeutic option.

The benefits of SGLT2 inhibitors on insulin resistance originate from multiple mechanisms. In patients with diabetes, Goto et al. evaluated the influence of empagliflozin treatment on insulin sensitivity of skeletal muscles; following a one-week regimen, noticeable improvement in insulin sensitivity was detected, which was attributed to a rapid correction of glucotoxicity [64]. Additional experimental and clinical studies have supported the benefits of SGLT2 inhibitors on β-cell function, insulin signaling, and insulin sensitivity via amelioration of glucotoxicity [65,66]. The effects of SGLT2 inhibitors on weight control and visceral fat lowering, attributed to glycosuria-related caloric deposition, are also crucial for insulin sensitivity. Studies in Japanese and Indian diabetic populations have demonstrated that SGLT2 inhibitors contribute to a reduction in body weight and visceral fat, improving insulin sensitivity via alleviation of lipotoxicity [67,68]. In animal models, SGLT2 inhibitors induced white adipose tissue browning and improved fat utilization via an M2 macrophage polarization-dependent mechanism, thereby increasing insulin sensitivity [69]. To maintain better glucose homeostasis and enhance insulin sensitivity, preservation of β-cell function is imperative, and SGLT2 inhibitors reportedly play vital roles in terms of this aspect. Robust clinical evidence [70,71] on the β-cell protection afforded by SGLT2 inhibitors has been documented, with several relevant mechanisms proposed, including deceleration of β cell death and regeneration of pancreatic islet cells via amelioration of glucotoxicity, lipotoxicity, inflammation, fibrosis, and oxidative damage [63,72]. Furthermore, Wei et al. reported that SGLT2 inhibitors induced β-cell self-replication, α-to-β cell conversion, and duct-derived β cell neogenesis in an animal model, partially mediated by the additional promotion of glucagon-like peptide-1 (GLP-1) secretion [73]. Finally, the attenuative effects of SGLT2 inhibitors on oxidative stress and inflammatory responses are also crucial for restoring insulin sensitivity, as discussed above.

In conclusion, SGLT2 inhibitors can decrease insulin resistance via multiple mechanisms, including glucotoxicity correction, caloric disposition and lipotoxicity modulation, β cell preservation, and attenuation of oxidative stress and inflammation. Collectively, the antioxidant properties and insulin-sensitizing effects of SGLT2 inhibitors could disrupt the interplay between oxidative stress and insulin resistance in T2DM and serve as favorable therapeutic options.

### 2.3. The Antioxidant Effects of SGLT2 Inhibitors on T2DM Outcomes

With favorable results reported in large clinical trials such as the Empagliflozin Cardiovascular Outcome Event Trial in Type 2 Diabetes Mellitus Patients-Removing Excess Glucose (EMPA-REG OUTCOME) trial, the Dapagliflozin in Patients with Heart Failure and Reduced Ejection Fraction (DAPA-HF) trial, the Dapagliflozin and Prevention of Adverse Outcomes in Chronic Kidney Disease (DAPA-CKD) trial, Canagliflozin Cardiovascular Assessment Study (CANVAS), and the Canagliflozin and Renal Events in Diabetes with Established Nephropathy Clinical Evaluation (CREDENCE) trial, the cardiorenal protective benefits of SGLT2 inhibitors have been widely recognized. Notably, clinical guidelines recommend SGLT2 inhibitors as the glucose-lowering agent for patients with diabetes presenting a higher cardiovascular risk or renal insufficiency [13,74]. Besides metabolic, hemodynamic, and diuretic mechanisms, the antioxidant and anti-inflammatory effects of SGLT2 inhibitors also contribute to their cardiorenal benefits [19]. Some clinical studies on SGLT2 inhibitors have directly assessed the link between antioxidant effects and their outcome benefits. In the Dapagliflozin Effectiveness on Vascular Endothelial Function and Glycemic Control (DEFENCE) trial, an open-label prospective blinded-endpoint study, patients with T2DM, previously treated with metformin, were randomized to receive either higher doses of metformin or metformin plus dapagliflozin; effects on endothelial function and oxidative stress, as determined by the flow-mediated dilation method and urinary 8-OHdG levels, respectively, were evaluated. After the 16-week study period, the dapagliflozin group demonstrated greater improvement in endothelial function, especially in patients with glycated hemoglobin (HbA1c) > 7.0%, and urinary 8-OHdG levels were significantly lower in the dapagliflozin group [75]. In another open-label prospective study evaluating Japanese patients with chronic heart failure and T2DM, the effects of add-on canagliflozin on fat distribution, cardiac natriuretic peptides, renal function, endothelial function, and oxidative stress were examined. During the 12-month treatment period, renal function, endothelial function, and cardiac natriuretic peptides were all improved, and a decline in oxidative stress indicated by ox-LDL was observed [76]. In summary, SGLT2 inhibitors afford additional cardiorenal protection in T2DM, possibly attributed to the antioxidant properties, as supported by experimental and clinical evidence.

### 2.4. The Possible Antioxidant Roles of SGLT2 Inhibitors in Type 1 Diabetes Mellitus (T1DM)

In addition to the beneficial effects of SGLT2 inhibitors in T2DM, there are increasing discussions about its possible adjuvant role in T1DM. In clinical studies, SGLT2 inhibitors demonstrate noticeably advantageous effects in T1DM, including lowering HbA1c and glucose variability, increasing time in optimal glucose range, as well as reduction in weight and insulin dose with little risk of hypoglycemia [77]. However, due to the risk of ketoacidosis, there are still controversies about the pros and cons of SGLT2 inhibitors in T1DM. Therefore, SGLT2 inhibitors have not been approved for T1DM in most countries [78]. There is a paucity of clinical research about the antioxidative benefits of SGLT2 inhibitors in T1DM. Its antioxidant roles are supported by some animal studies. In a mouse model study of T1DM, empagliflozin displayed a beneficial effect on preserving β-cell, probably via protection of pancreatic β-cell from glucotoxicity-induced oxidative stress [72]. Tahara et al., also reported that ipragliflozin reduced liver levels of oxidative stress biomarkers and ameliorated hepatic steatosis in a rat model of T1DM [26]. More studies are required to clarify the role of SGLT2 inhibitors in T1DM and its clinical antioxidant beneftis as well as mechanisms in this population.

### 2.5. Summary of SGLT2 Inhibitors as Antioxidants in Diabetes

**Figure 2:**
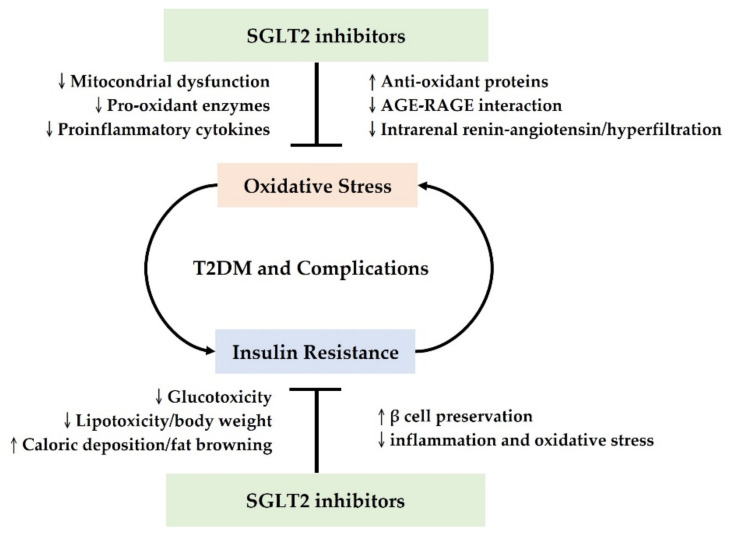
Schematic diagram presenting mechanisms via which SGLT2 inhibitors mediate antioxidant effects against T2DM and its complications. AGE, advanced glycation end product; RAGE, receptor for advanced glycation end product; SGLT2, Sodium-glucose cotransporter 2; T2DM, type 2 diabetes mellitus; ↑, increase; ↓, reduce.

The antioxidant effects of SGLT2 inhibitors in T2DM are mediated by multiple mechanisms and contribute to several beneficial effects, supported by robust experimental and clinical research. Based on the detailed discussion described above, we conclude that SGLT2 inhibitors are able to reduce oxidative stress in T2DM by upregulating antioxidant proteins, downregulating pro-oxidant enzymes, reducing AGE-RAGE interactions, improving mitochondrial function, reducing proinflammatory cytokines and inhibiting the intrarenal RAS. The reduced oxidative stress leads to improved insulin resistance. Complementary to the antioxidant roles, SGLT2 inhibitors can improve insulin resistance by reducing glucotoxicity and lipotoxicity, increasing caloric deposition and attenuating inflammation. This conclusion is illustrated in Figure 2 and the preclinical and clinical studies supporting the antioxidant roles of SGLT2 inhibitors in diabetes are summarized in Table 1.

## 3. SGLT2 Inhibitors as Antioxidants in Heart Diseases

In patients with diabetes, aggressive blood sugar control can help prevent or improve small vessel disease (neuropathy, nephropathy, retinopathy), as well as delay the occurrence of nephropathy [79]. However, in diabetic patients with significant vascular diseases, including stroke, myocardial infarction, heart failure, and death due to cardiovascular disease, active blood sugar control may fail to reduce the risk of occurrence [79]. Previously, in addition to metformin, which may reduce myocardial infarction and heart failure, drugs such as sulfonylurea, thiazolidinedione, dipeptidyl peptidase 4 inhibitors, and glucagon-like peptide-1 receptor agonists may increase the incidence of heart failure or present inconclusive results (no class effect) [80,81,82,83,84,85,86]. In contrast to previous oral glucose-lowering agents, SGLT2 inhibitors showed additional benefits in cardiovascular outcomes in three influential studies: the Dapagliflozin Effect on Cardiovascular Events-Thrombolysis in Myocardial Infarction 58 (DECLARE-TIMI 58) trial, the EMPA-REG OUTCOME trial, and the CANVAS program [8,12,87]. Mounting evidence has revealed that the cardiac benefits of SGLT2 inhibitors may be independent of glycemic control. The DAPA-HF trial and cardiovascular and renal outcomes with empagliflozin in heart failure (EMPEROR-Reduced) trial demonstrated that among patients with heart failure and a reduced ejection fraction, the risk of worsening heart failure or cardiovascular death was lower in those who received SGLT2 inhibitors than in those who received the placebo, regardless of the presence or absence of diabetes [7,88]. In this review, we focus on the relationship between SGLT2 inhibitors and oxidative stress in heart failure and discuss the potential antioxidant role of SGLT2 inhibitors.

### 3.1. Oxidative Stress, Diabetic Cardiomyopathy and Heart Failure

Heart failure and diabetes both present chronic low-grade inflammation. Cellular ion dysregulation, AMPK inactivation, and ROS production are relevant factors in the development of inflammation [89]. Inflammation may also lead to fibrosis, cell death, and cardiac remodeling. Hence, inhibiting inflammation may be a crucial mechanism for preventing and treating diabetes-related heart failure. Early increased oxidative stress is prominent in heart failure and T2DM pathogenesis [90,91]. Highly reactive ROS can oxidize several proteins, thereby altering the function of these proteins. Furthermore, ROS is an upstream driver of endothelial dysfunction associated with heart failure by increasing the uncoupling of nitric oxide synthase to convert nitric oxide into peroxynitrite [92,93,94,95]. ROS have several detrimental effects on the myocardium, inducing cardiomyocyte electrophysiologic and contractile dysfunction, mitochondrial dysfunction, and increased cardiac fibrosis [96]. Therefore, reducing ROS normalizes the function of many proteins in cardiomyocytes, which may prevent the development of heart failure.

Both heart failure and diabetic cardiomyopathy result in an energy crisis, which is manifested by a decrease in the cardiac phosphocreatine (PCr) to adenosine triphosphate (ATP) ratio. Accordingly, cell low-energy sensors, including sirtuin 1 (SIRT1) and AMPK, are activated. SIRT1 and AMPK are activated not only by starvation but also by cellular stressors, including hypoxia, ROS, injured organelles, and misfolded proteins [97]. SIRT1 eliminates oxidative stress by enhancing antioxidant activity, directly reduces the inflammatory response to oxygen free radicals, and reduces the lethality of oxidative stress [98]. AMPK maintains mitochondrial function, thereby reducing ROS formation and attenuating proinflammatory and pro-apoptotic responses [99]. AMPK activity is increased in failed cardiomyocytes [100]. AMPK acts as an energy sensor and activates catabolism by increasing glucose uptake and glycolysis while impairing anabolic reaction [101]. Targeting the AMPK pathway by increasing AMPK activity has been shown to combat cardiac hypertrophy and myocardial failure [102]. In addition, AMPK activity can be associated with non-metabolic cellular processes, including regulation of vascular tone and inhibition of inflammation [101,103,104].

AGEs mainly originate from the rearrangement of early glycation products [105]. AGEs accumulate in plasma and vascular tissues and directly interact with the extracellular matrix to induce arterial stiffness and reduced elasticity [106]. RAGEs on the surface of endothelial cells, vascular smooth muscle cells, and monocytes promote oxidative stress and cause inflammation and fibrosis of the vessel [107,108]. Activation of AGE-RAGE signaling is associated with an increased risk of acute coronary syndrome and heart failure [109].

### 3.2. The Potential Antioxidant Effects of SGLT2 Inhibitors in Heart Failure

Several mechanisms have been proposed for the cardiovascular effects of SGLT2 inhibitors, including reduced blood pressure, cardiac preload and afterload, plasma volume, increased hematocrit, increased myocardial energetic efficiency, inhibition of Na_+_/H_+_ exchanger (NHE), and decreased oxidative stress [1,20,110]. This review highlights the potential antioxidant effects of SGLT2 inhibitors in heart failure.

Notably, the effect of SGLT2 inhibitors in stimulating the activity of low-energy sensors is not mediated by the SGLT2 protein in cells, as this effect can also be observed in organs that do not express SGLT2 [37,111]. SGLT2 inhibitors activate SIRT1/AMPK and inhibit AKT/mTOR signaling; therefore, they can promote autophagy beyond its effect on glucose or insulin [111,112,113]. In addition, the activation of SIRT1/AMPK and inhibition of AKT/mTOR signal transduction mediated by SGLT2 inhibitors can result in reduced oxidative stress, normalization of mitochondrial structure and function, inhibition of inflammation, reduction of coronary microvascular damage, enhancement of contractility, and reduced incidence of cardiomyopathy [114]. Furthermore, the activation of AMPK/SIRT1 and autophagic flux can be associated with the downregulation of ion exchangers, reportedly involved in the pathogenesis of diabetic cardiomyopathy [115]. Finally, the enhancement of HIF-1α/HIF-2αsignaling by SGLT2 inhibitors may amplify the autophagic flux enhanced by AMPK/SIRT1, which may be an important contribution to the cardiac benefits of these drugs, which are not observed with other glucose-lowering agents [116].

Reportedly, reduced AGE production and inhibition of the AGE-RAGE axis, along with the parallel reduction of oxidative stress following ipragliflozin treatment, improved the endothelial function of diabetic mice [117]. Empagliflozin improved the cardiac diastolic function in a female rodent model of diabetes without normalizing myocardial AGE levels [118]. In cultured H9C2 cells, a hypoxia/reoxygenation model, empagliflozin increased cell viability and maintained ATP levels [119]. These effects were equally present in myocytes stimulated by AGE, and empagliflozin did not alter the expression level of RAGE; this suggested that these pro-survival mechanisms of empagliflozin were not mediated through AGE/RAGE signaling. The importance of AGEs and AGE-RAGE signaling as mediators of SGLT2 inhibitor effects in the human heart remains elusive.

In patients with diabetes, hyperglycemia can promote glucose uptake by cardiomyocytes, which in turn impairs cardiac function [120]. It has been reported that glucose toxicity increases cardiac oxidative stress and exacerbates myocardial injury in patients with T2DM [121]. SGLT2 inhibitors can prevent the absorption of excess glucose by the heart. These inhibitors enhance the β-hydroxybutyrate content and convert the energy supply from fatty acids and glucose to ketones, thus increasing the metabolic efficiency of the myocardium and decreasing oxygen consumption [122]. A study using a non-diabetic porcine model with myocardial infarction revealed that empagliflozin increased myocardial ketone consumption while simultaneously decreasing glucose consumption. Increased myocardial energetics leads to reversed anatomical, metabolic, and neurohormonal remodeling [46]. Although animal studies have confirmed this hypothesis, supportive clinical data are still lacking. In an attempt to translate these preclinical results into the clinical arena, a recent clinical trial using empagliflozin (EMPA-TROPISM trial) was conducted in non-diabetic patients with heart failure [123].

In summary, several lines of evidence emphasize the antioxidant roles of SGLT2 inhibitors in cardiovascular diseases. Although this evidence supports the cardioprotective effects of SGLT2 inhibitors observed in large clinical trials, additional research is critical to further elucidate regulatory pathways and explore novel therapeutic targets that may present synergistic effects with SGLT2 inhibitors in affording cardioprotection.

## 4. SGLT2 Inhibitors as Antioxidants in Nephropathies

### 4.1. Clinical Evidence Demonstrating Antioxidant Capacities of SGLT2 Inhibitors in Diabetic Kidney Disease

The redox status, as well as the balance between oxidative stress and the antioxidant defense system, have been recognized as critical mechanisms leading to the progression of diabetic kidney disease. Emerging clinical observations have suggested the potential of SGLT2 inhibitors in modulating the redox state in kidney diseases. In patients with T2DM and microalbuminuria, 12 weeks of canagliflozin treatment significantly reduced the urinary albumin level, urinary 8-OHdG and urinary L-fatty acid-binding protein (L-FABP) [124]. In a randomized clinical trial assessing 80 patients with inadequately controlled T2DM, 16 weeks of dapagliflozin add-on therapy to metformin improved endothelial function and oxidative stress (reduced urine 8-OHdG) [75]. An acute effect on the kidney was indicated by a 2-day treatment with dapagliflozin in patients with T2DM, whose oxidative stress (decline in urinary isoprostanes) and endothelial function (including renal resistive index) improved independently of changes in blood pressure [125].

### 4.2. SGLT2 Inhibitors Reduce Renal Oxidative Stress in Preclinical Studies

SGLT2 inhibitors may protect kidney tissues against oxidative damage by reducing ROS, along with improvements in albuminuria and hyperfiltration. Animal studies have supported the effect of SGLT2 inhibition in reducing oxidative stress by modifying pro-oxidant activity. In one study utilizing db/db mice, treatment with dapagliflozin suppressed renal oxidative stress, glomerular mesangial matrix accumulation, interstitial proinflammatory macrophage infiltration, and fibrosis [25]. Interestingly, the generated ROS and Nox4 expression were predominantly localized in the renal interstitium. In a different model with BTBR ob/ob diabetic mice, investigators observed that an elevation of tricarboxylic acid cycle metabolites from high glucose levels resulted in accelerated oxidative phosphorylation and increased oxidative stress. Ipragliflozin attenuated oxidative stress in the kidney cortex, especially in the glomeruli, and improved albuminuria, hyperfiltration, and mesangial expansion. However, tubulointerstitial inflammation with macrophage infiltration was not decreased by ipragliflozin in this model [126].

### 4.3. Antioxidant Mechanisms of SGLT2 Inhibitors in Affording Renoprotection

AGEs and RAGE, stimulated by hyperglycemia, can lead to ROS production and play an important role in diabetic nephropathy. In streptozotocin-induced diabetic rats, empagliflozin significantly decreased the renal expression of AGEs, RAGE, 8-OHdG, urinary 8-OHdG, and L-FAB. Moreover, empagliflozin treatment suppressed the activation of inflammatory and fibrotic gene expression [35]. Similar findings regarding the AGE-RAGE axis were observed in human proximal tubular cells [127].

Mitochondrial dysfunction plays a critical role in kidney disease. A recent transcriptomic analysis of kidney biopsies collected from patients with diabetic kidney disease has suggested that dapagliflozin treatment may exert its renoprotective effect by modifying molecular pathways related to energy metabolism, mitochondrial function, and endothelial function [128]. In high-fat diet (HFD)-fed mice, ipragliflozin treatment reversed kidney tubular mitochondrial damage and in HK-2 cells, ipragliflozin restored GTPase levels to their normal values [129]. In streptozotocin-induced diabetic mice, empagliflozin decreased mitochondrial fragmentation. Additionally, a study evaluating human renal proximal tubular cells showed improvements in mitochondrial biogenesis and balanced fusion-fission protein expression, increased autophagic activity, decreased mitochondrial ROS production, and reduced apoptotic and fibrotic protein expression. Moreover, empagliflozin restored AMPK-α phosphorylation and normalized the AMP-to-ATP ratio levels [16]. SGLT2 inhibitors are postulated to enhance SIRT1, AMPK, and HIF-2α signaling, leading to improved mitochondrial and peroxisomal functions and reduced oxidative stress. These findings, along with improved autophagic flux, may ameliorate the glomerular hyperfiltration, podocyte dysfunction, and tubulointerstitial inflammation observed in preclinical and clinical studies [130].

The processes of oxidative stress and inflammation are elaborately interconnected. As illustrated in rats with renal oxidative damage induced by isoprenaline, canagliflozin treatment reduced oxidative stress and restored depleted antioxidants by activating antioxidant/anti-inflammatory signaling pathways (AMPK, AKT, and eNOS), and inhibiting inducible NOS (iNOS) and NOX4 [131]. As the redox state is a delicate and dynamic balance between oxidative and antioxidant systems, other studies have explored these two systems simultaneously. In streptozotocin-induced diabetic rats, SGLT2 inhibition by phlorizin reduced nitrogen free radical species (3-nitrotyrosine (3-NT) in the kidney, while restoring the antioxidant activities of catalase (CAT) and glutathione peroxidase (GPx) [29]. Furthermore, antioxidant effects have been documented in studies where SGLT2 inhibition reduced oxidative stress by restoring manganese/copper/zinc-dependent SODs and catalase expression in renal tissues of diabetic animals [39,132]. A plethora of cellular evidence supports that SGLT2 inhibition reduces renal oxidative stress and inflammation. In cultured human proximal tubular cells exposed to high glucose, tofogliflozin suppressed oxidative stress, MCP-1 gene expression, and apoptosis [22]. In cultured mouse renal proximal tubular cells, dapagliflozin suppressed the high glucose-induced gene expression of inflammatory cytokines and oxidative stress. These data indicate that dapagliflozin ameliorates diabetic nephropathy by improving hyperglycemia and inhibiting inflammation and oxidative stress [25]. Canagliflozin attenuated high-glucose-induced ROS overproduction in cultured mouse mesangial cells by inhibiting the activation of the protein kinase C/NADPH oxidase pathway [133].

## 5. SGLT2 Inhibitors as Antioxidants to Treat Fatty Liver Disease

Liver diseases, particularly nonalcoholic fatty liver disease (NAFLD), are closely associated with T2DM. NAFLD frequently occurs in obese individuals with insulin resistance and can progress to nonalcoholic steatohepatitis, fibrosis, and cirrhosis [134]. Oxidative stress [135,136,137] and inflammation [138] are important factors mediating hepatocyte injury in NAFLD. However, not all glucose-lowering agents are beneficial in NAFLD. Only pioglitazone [139] and glucagon-like peptide receptor agonists [140] effectively reduce the liver fat content. As SGLT2 inhibitors improve insulin resistance, glucotoxicity, and lipotoxicity, their therapeutic potential in T2DM related liver complications can be expected.

### 5.1. Effects of SLGT2 Inhibitors on Serum Liver Enzymes

Compared with metformin, glimepiride, and dipeptidyl peptidase-4 inhibitors, SGLT2 inhibitors are associated with lower liver enzyme levels [141], regardless of the co-existence of NAFLD. Among the liver enzymes, alanine aminotransferase (ALT), and gamma-glutamyl transferase (GGT) levels are significantly reduced, but aspartate aminotransferase (AST) levels are less consistent [142]. Notably, the beneficial effect of SGLT2 inhibitors on liver enzyme levels was associated with baseline ALT levels. Reductions in serum liver enzymes may appear small and statistically insignificant if the study population presents normal liver enzyme levels. The SGLT2 inhibitor-related reduction in liver enzymes was most significant in the highest ALT tertile [143]. In a cohort study that analyzed the electronic medical records database, the authors revealed that the higher the baseline ALT level, the greater the decline after SGLT2 inhibitor treatment [144]. In another meta-analysis, the effects of canagliflozin on liver enzymes were analyzed at different doses; reduced liver enzymes seemed to demonstrate a dose-response effect [145].

### 5.2. Effects of SLGT2 Inhibitors on Fatty Liver and Fibrosis

Fibrosis 4 (FIB-4) index and transient elastography (FibroScan) are commonly employed as non-invasive examinations to analyze the severity of liver fibrosis and steatosis. In a randomized controlled study comparing the efficacy of ipragliflozin and pioglitazone on NALFD with T2DM, only ipragliflozin significantly reduced the FIB-4 index, although both drugs significantly decreased HbA1c and liver enzymes. Interestingly, the impact of ipragliflozin and pioglitazone on body weight and the subcutaneous fat area differed completely [139]. A reduction in FIB-4 was also observed in other SGLT2 inhibitor trials. Itani et al. demonstrated that canagliflozin effectively reduced the FIB-4 index in patients with NALFD [146]. A similar response was observed in another study using empagliflozin [147]. However, in a post hoc pooled analysis of ertugliflozin clinical trials (N = 4859), no change in the FIB-4 index was observed in the ertugliflozin group when compared with the non-ertugliflozin group at 52 weeks [148]. More recently, Seno et al. examined the efficacy of luseogliflozin in NALFD [149]. The FIB-4 index was unaltered after 52 weeks of treatment, and the results were comparable with those of the LEAD trial [150].

Using FibroScan, Shimizu et al. compared the effect of dapagliflozin with other standard oral glucose-lowering agents on hepatic steatosis and fibrosis in patients with T2DM presenting NAFLD. After 24 weeks of treatment, patients in the dapagliflozin group demonstrated significantly decreased AST, ALT, and GGT levels when compared with the control group. The controlled attenuation parameter, a marker of liver steatosis, was significantly reduced by 8% in the dapagliflozin group; however, the improvement in liver stiffness measurement did not reach statistical significance (from 9.49 kPa to 8.01 kPa, *p* = 0.0539). On dividing patients according to the severity of liver stiffness, dapagliflozin decreased liver stiffness measurement from 14.7 kPa at baseline to 11.0 kPa (*p* = 0.0158) in patients with higher baseline liver fibrosis scores (≥8 kPa) [151]. A recent propensity score-matched study revealed that SGLT2 inhibitors (including six kinds of drugs) reduced the controlled attenuation parameter by 10% and liver stiffness measurement by 26% (both *p* < 0.001) at 48 weeks [152]. In this study, improvement in liver stiffness measurement was independent of changes in body weight or HbA1c [152]. The potential of SGLT2 inhibitors to provide beneficial effects in non-diabetic patients with NAFLD remains unknown. Taheri et al., reported that empagliflozin significantly reduced both the controlled attenuation parameter and liver stiffness measurement. However, the placebo group showed a significant improvement in the controlled attenuation parameter and liver stiffness measurement. Furthermore, no significant difference was observed between the SGLT2 inhibitor and placebo groups after 24 weeks of treatment [153].

### 5.3. Effects on Biopsy-Proven Histopathological Abnormalities

Akuta et al., first evaluated the usefulness of canagliflozin for histopathological changes in NAFLD, revealing that canagliflozin improved steatosis and NAFLD activity scores in all five patients [154]. Scores of steatosis, lobar inflammation, ballooning, and fibrosis decreased by 78%, 33%, 22%, and 33%, respectively, at 24 weeks when compared with baseline values [155]. The same group of researchers further reported that canagliflozin exerted a favorable outcome on NAFLD during long-term treatment (≥1 year). Among seven patients, six presented liver histopathological improvements despite worsening body mass index and waist circumference at the final biopsy when compared with 24-week findings [156].

### 5.4. Antioxidant and Anti-Inflammation Capacities of SGLT2 Inhibitors in NAFLD

The pathogenesis of NAFLD is multifactorial. The “first hit” for NAFLD development begins with genetic, metabolic, and environmental factors that work in concert to promote the accumulation of highly toxic free fatty acids in hepatocytes, along with the increased de novo hepatic fatty acid synthesis from glucose. NAFLD progression involves the occurrence of “parallel, multiple-hit” injuries, such as oxidative stress, mitochondrial dysfunction, endoplasmic reticulum stress, increased production of adipokines and myokines, the endotoxin-induced release of inflammatory cytokines, and iron overload. These factors activate various signaling cascades leading to inflammation, cell death, and fibrosis in the liver [135,157,158,159].

Accumulated clinical evidence, even in small human studies, has revealed that SGLT2 inhibitors are beneficial in normalizing liver enzymes, reducing fatty liver, and attenuating fibrosis. The ability of SGLT2 inhibitors to improve insulin resistance, glucotoxicity, and lipotoxicity may account for the improved metabolic factors in the “first hit” for NAFLD development. Several lines of evidence from preclinical studies have demonstrated the crucial roles of SGLT2 inhibitors in combating the “multiple-hit” pathogenesis of NAFLD, especially in reducing oxidative stress and inflammation. In db/db mice, dapagliflozin reduced ALT and liver lipid accumulation and fibrosis. These benefits were associated with reduced hepatic production of myeloperoxidase (MPO) and ROS [160]. In western diet-fed ApoE knockout mice, empagliflozin treatment reduced hepatic lipid droplet accumulation and downregulated expression levels of tumor necrosis factor (TNF), interleukin (IL)-6, and MCP-1 when compared with the control or glimepiride groups [36]. Tahara et al. reported that ipragliflozin significantly reduced liver oxidative stress biomarkers (thiobarbituric acid-reactive substances [TBARS] and protein carbonyl groups) and plasma inflammation markers (IL-6, TNF-α, MCP-1, and CRP) in rats [26]. Similar findings were reported by Nakano et al., who demonstrated that remogliflozin reduced hepatic mRNA levels of TNF-α, MCP-1, TBARS, and oxygen radical absorbance capacity in HFD-fed mice [161]. Canagliflozin reduced hepatic oxidative stress, as reflected by the decreased hepatic malondialdehyde (MDA) levels. Furthermore, canagliflozin enhanced the antioxidant enzyme activity, as reflected by the increased SOD and glutathione peroxidase (GPx) activity [162]. In primary mouse hepatocytes, canagliflozin activated AMPK by inhibiting complex I of the respiratory chain. Activated AMPK facilitates acetyl-CoA carboxylase (ACC) phosphorylation and ultimately leads to reduced lipogenesis [163]. In HFD-induced obese mice, a novel SGLT2 inhibitor, NGI001, suppressed hepatic lipid accumulation and inflammation. In addition to observations in mouse cells, NGI001 ameliorated fat deposition by increasing phosphorylation of AMPK and ACC in human hepatocyte HuS-E/2 cells. This cascade eventually led to the upregulation of downstream β-oxidation-associated molecules and downregulation of downstream fatty acid synthesis-related molecules [164]. Table 2 summarizes the evidence from preclinical studies demonstrating SGLT2 inhibitors as antioxidants in NAFLD.

## 6. SGLT2 Inhibitors as Antioxidants in Neural Disorders

Glucose is the primary metabolic substrate and an essential source of normal neural function [165]. It is transported into the brain across the blood-brain barrier (BBB) and made available to neurons and glial cells via specific transporters. Glucose transporters (GLUTs) comprise two major groups: 1) GLUT-independent facilitated transporters [166] and 2) SGLTs. GLUT1 is expressed in the BBB and glial cells, whereas GLUT3 is expressed in neurons [167]. SGLTs were initially found to occur in the kidneys and intestines [168,169]. Recently, it was reported that SGLTs are widely expressed in the brain, including the frontal cortex, hippocampus, caudate nucleus, putamen, parietal cortex, and paraventricular nucleus of the hypothalamus. However, SGLTs are poorly expressed in the brainstem [170].

SGLT2 inhibitors are partially lipid-soluble and can cross the BBB. Several studies have confirmed the presence of SGLT2 in the brain, emphasizing the potential therapeutic roles of SGLT2 inhibitors in neurological diseases [171,172].

### 6.1. SGLT2 Inhibitors Attenuate Cerebral Oxidative Stress in Obesity or Diabetic-Induced Cognitive Impairment

Metabolic disorders, including obesity and diabetes, are major risk factors for cardiovascular diseases and cognitive dysfunction [173]. Lin et al., reported that empagliflozin significantly ameliorated cognitive dysfunction in db/db mice, associated with the attenuation of cerebral oxidative stress and an increase in cerebral brain-derived neurotrophic factor [174]. In addition, Hierro-Bujalance et al. reported that empagliflozin treatment reduced the microglial burden in the parenchyma of db/db mice and APP/PS1xdb/db mice [175].

### 6.2. SGLT2 Inhibitors Suppress Cerebral Oxidative Stress in Stroke

Cerebral ischemic stroke is the second leading cause of mortality and long-term disability worldwide [176]. Hyperglycemia, hypertension, dyslipidemia, and obesity are important risk factors for stroke. Several studies have demonstrated improvements in these parameters in diabetic and obese murine models following treatment with SGLT2 inhibitors.

Stroke can be prevented by eliminating risk factors, including carotid atherosclerosis, a chronic inflammation of the blood vessels that causes plaque formation and subsequent arterial narrowing [177]. Abdel-Latif et al. reported that empagliflozin ameliorated neurological defects in a rat model of ischemic stroke by utilizing occlusion of the carotid arteries [178]. Notably, the beneficial effect of empagliflozin was dose-dependent. To prevent ischemia-induced hypoxia, HIF-1α is upregulated to restore oxygen homeostasis. Compared with the control group, empagliflozin administration increased the levels of HIF-1α and vascular endothelial growth factor, decreased expression levels of caspase-3, and limited the infarct volume.

Amin et al., demonstrated that, compared with gliclazide, empagliflozin decreased the infarct volume and suppressed cerebral oxidative stress, inflammation, and markers of apoptosis in the rat brain [179]. Empagliflozin decreased MDA, increased catalase activity, and elevated GSH levels in brain tissues [179]. These data suggested that empagliflozin can reduce oxidative stress in stroke. However, the EMPA-REG OUTCOME trial revealed no significant difference in stroke between empagliflozin- and placebo-treated groups [180]. In the CANVUS trial, no significant difference in stroke was observed between canagliflozin- and placebo-treated groups [8].

### 6.3. Therapeutic Implications of SGLT2 Inhibitors in Alzheimer’s Disease and Parkinson’s Disease

Alzheimer’s disease (AD) and Parkinson’s disease (PD) are the most common age-related neurodegenerative disorders. AD is the sixth leading cause of mortality, consisting of over two-thirds of all reported cases of dementia. By 2050, the global incidence of AD is expected to grow to 106.8 million [181]. PD is primarily a movement disorder characterized by bradykinesia, hypokinesia, and akinesia, along with resting tremor and muscle rigidity. Currently available medications for AD and PD afford symptomatic relief but cannot halt the underlying neurodegenerative process. Therefore, it is worth examining whether SGLT2 inhibitors have therapeutic potential in AD and PD.

The major pathological feature of AD is the accumulation of extra- and intra-cellular residues called plaques, composed of beta-amyloid and neurofibrillary tangles (NFTs). Recent studies have suggested that SGLT inhibition may have a therapeutic effect on this process. By employing the APP/PS1xdb/db model, Hierro-Bujalance et al., reported that empagliflozin reduced senile plaque density, with an overall reduction in soluble and insoluble amyloid β levels in the cortex and hippocampus of empagliflozin-treated mice [175]. In a scopolamine-induced memory impairment rat model, Arafa et al., observed that another SGLT2 inhibitor, canagliflozin, can prevent memory impairment [182]. The authors attributed this effect to possible acetylcholinesterase inhibitory ability as an additional property of SGLT2 inhibitors.

Oxidative stress-induced glial cell activation, neuroinflammation, and mitochondrial dysfunction lead to various molecular events in brain neurons, causing neuronal cell death in both AD and PD. A recent study by Arab et al. reported the promising role of dapagliflozin in the management of PD. In the well-recognized rotenone-induced PD rat model, the authors revealed that dapagliflozin improved PD motor function and improved motor coordination in the open-field and rotarod tests. Through a series of investigations, Arab et al. further documented that dapagliflozin alleviated neuronal oxidative stress by lowering lipid peroxides. Furthermore, they demonstrated that dapagliflozin counteracted ROS-dependent neuronal apoptosis and upregulated glial cell-derived neurotrophic factor (GDNF) and its downstream PI3K/AKT/GSK-3β (Ser9) pathway [183].

### 6.4. Applications of SGLT2 Inhibitors in Epilepsy

Over 50 million people live with epilepsy, and approximately 10 million reportedly develop drug-resistant epilepsy. Erdogan et al., demonstrated that dapagliflozin decreased seizure activity in rats with pentylenetetrazol-induced seizures [184]. The authors postulated that the anti-seizure effects could be attributed to decreased glucose availability and a reduction in sodium transport across neuronal membranes, which enable a stabilizing effect against excitability and depolarization.

### 6.5. Summary of the Antioxidant Role of SGLT2 Inhibitors in Neural Diseases

Collectively, the effects of SGLT2 inhibitors on diabetes-induced cognitive impairment and stroke may be attributed to the systemic effects of glycemic control, anti-inflammation, and antioxidative effects. In addition, SGLT2 inhibitors may penetrate the BBB to exert their antioxidative effects on the brain tissue. In AD, SGLT2 inhibitors may reduce senile plaques, and canagliflozin may possess additional acetylcholinesterase inhibitory ability. In a rat PD model, dapagliflozin reportedly attenuated neuronal injury and motor dysfunction by targeting ROS-dependent AKT/GSK-3β/NF-κB and DJ-1/Nrf2 pathways. In epilepsy, dapagliflozin may stabilize seizure activity by reducing glucose and sodium transport into neurons. These promising findings are summarized in Table 3. Further clinical studies are warranted to confirm these findings.

## 7. SGLT2 Inhibitors Act as Potent Anti-Cancer Agents

ROS are closely associated with cancer progression. Reportedly, tumor cells present elevated ROS levels when compared with non-transformed normal cells [185]. High levels of oncogenic ROS can induce lipid/protein/DNA damage, thus promoting genomic instability and tumorigenesis [186]. Although ROS may contribute to genome instability, the potential roles of ROS as signaling molecules that promote tumor cell viability, growth, neovascularization, and metastasis have emerged [187]. Thus, potent antioxidants may act as chemopreventive or therapeutic agents for cancer management.

It has been demonstrated that SGLT2 inhibitors can serve as potent antioxidants that afford protection against oxidative stress-induced tissue injury, not only via their glucose-lowering effects but also by either reducing free radical production or by modulating the antioxidant system [27]. Several preclinical and clinical studies have indicated that SGLT2 inhibitors (canagliflozin, dapagliflozin, and empagliflozin) show anti-cancer activity in many types of cancers, including hepatocellular carcinoma (HCC) [33,188,189,190], pancreatic cancer [191], squamous cell carcinoma [192], renal cell carcinoma [193], prostate cancer [34], cervical cancer [194], lung cancer [30,34], breast cancer [195] and colon cancer [196].

**Figure 3:**
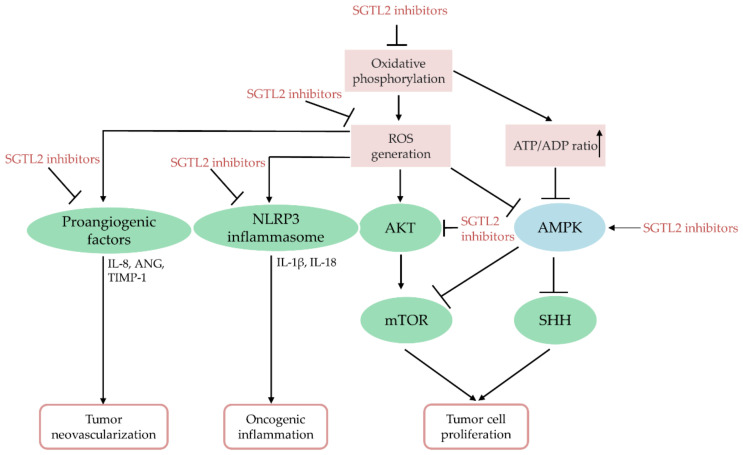
The proposed mechanisms of SGLT2 inhibitors against tumor development. ANG, angiogenin; IL-8, interleukin-8; NLRP3, NOD-like receptor family pyrin domain-containing 3; SHH, sonic hedgehog; TIMP-1, tissue inhibitors of metalloproteinase-1.

ROS are potent signal transducers with multiple biological functions, participating in tumor cell growth, cell survival, energy metabolism, oncogenic inflammation, and neovascularization [197]. Reportedly, canagliflozin can inhibit mitochondrial complex I-supported respiration, thus arresting cell growth via AMPK activation in prostate and lung cancer cells [34]. In breast and cervical cancers, dapagliflozin and empagliflozin inhibited mTOR and sonic hedgehog signaling via AMPK activation to retard tumor cell growth, respectively [194,195]. Oxidative stress causes AMPK protein oxidation and inactivation, as well as induces PTEN oxidation and inactivation to activate AKT signaling [198]. It has also been reported that canagliflozin treatment can repress AKT phosphorylation and activity in HCC cells [33] and squamous cell carcinomas [192]. Free radicals are potent angiogenesis inducers in human diseases such as cancer [199]. Canagliflozin administration reduced the angiogenic function of HCC by downregulating the proangiogenic factors IL-8, angiogenin, and tissue inhibitors of metalloproteinase-1 [33]. ROS-induced chronic inflammation is a promoter of cancer initiation and tumorigenesis [40,200]. Dapagliflozin or empagliflozin can inhibit the activity of the ROS-induced NOD-like receptor family pyrin domain-containing 3 (NLRP3) inflammasome [201,202]. Furthermore, canagliflozin and tofogliflozin demonstrated anti-inflammatory and chemopreventive effects in nonalcoholic steatohepatitis- and diethylnitrosamine-induced HCC models, respectively [188,189,190]. Collectively, SGLT2 inhibitors may act as potent anti-cancer agents by blocking ROS-induced cell growth, angiogenesis, and chronic inflammation in multiple cancers. Figure 3 presents a summary of the effects mediated by SGLT2 inhibitors on tumor progression.

It has been reported that SGLT2 inhibition only causes ~60% glucose excretion, an effect mediated by kidney SGLT1 upregulation [203]. Sotagliflozin, a dual SGLT1/2 inhibitor, has been approved to treat T1DM by FDA [204]. SGLT1 is also overexpressed in prostate, breast, skin, colon and ovarian cancer cells [205,206]. SGLT1 and epidermal growth factor receptor (EGFR, an oncoprotein) interaction can promote protein stability and activity each other in cancer cells [205,207,208]. Besides, SGLT1/EGFR interaction also affects the sensitivity of radiotherapy due to chromatin remodeling [209]. Some studies also indicate that SGLT1 targeting agents (Phlorizin or RNAi) may act as a novel adjuvant therapy for cancer treatment via the disruption of SGLT1/EGFR interaction [208,209,210]. Moreover, SGLT1 inhibition can inhibit diabetes-induced ROS formation, and dual SGLT-1/2 inhibition by sotagliflozin treatment also shows cardioprotective effect by its antioxidative function [211]. Thus, SGLT1 targeting or dual SGLT1/2 inhibition may also serve as novel anti-cancer strategies due to ROS reduction and the disruption of SGLT1/EGFR interaction.

## 8. Conclusions and Perspectives

Oxidative stress is a major cause of numerous human diseases, including diabetes, cardiovascular diseases, nephropathy, NAFLD, neural disorders, and cancers. Underlying mechanisms include mitochondrial dysfunction, AGE production, pro-oxidant enzyme activation, and hemodynamic alterations. Our review provides updated evidence that SGLT2 inhibitors exert antioxidant effects in common and important human diseases. As a novel class of glucose-lowering agents, SGLT2 inhibitors act as indirect antioxidants, as they can reduce high glucose-induced oxidative stress. Furthermore, SGLT2 inhibitors demonstrate therapeutic advantages directly by scavenging free radicals and boosting biological antioxidant systems.

Although the antioxidant roles of SGLT2 inhibitors have been better characterized in diabetes, cardiovascular diseases, and nephropathies, further research is required to elucidate the precise pathogenesis of these diseases and explore novel therapeutic targets that may afford synergistic effects with SGLT2 inhibitors. Furthermore, our review presents promising therapeutic implications of SGLT2 inhibitors as antioxidants in NAFLD, neural disorders, and cancers. Investigations assessing the association between SGLT2 inhibitors and known signaling pathways that regulate mitochondrial function and redox homeostasis in diabetes, as well as in the heart and kidneys, could be future research directions for understanding the progression of NAFLD to fibrosis. Studies on the antioxidant roles of SGLT2 inhibitors in neural disorders are relatively few and are urgently required. Most current medications afford symptomatic relief but fail to stop the progression of neural disorders. Our review reveals the therapeutic potential of SGLT2 inhibitors in neural disorders and highlights the research gap. In addition, by characterizing the antioxidant effects of SGLT2 inhibitors in cancers, our review could be valuable in developing promising cancer treatments.

## Figures and Tables

**Table 1:** Preclinical and clinical studies supporting the antioxidant roles of SGLT2 inhibitors in diabetes.

SGLT2 Inhibitor	Study Subject	Findings	References
Empagliflozin	Rats	↓Mitochondrial dysfunction	[59]
Empagliflozin	Rats	↓AGE-RAGE signaling	[24]
Empagliflozin	Mice	Regulate Sestrin2-mediated AMPK-mTOR signaling	[56]
Dapagliflozin	Mice	↓NOX4 &↑Nrf2-HO-1-mediated signaling	[25]
Phlorizin	Human endothelial cells	↑PI3K/AKT/eNOS signaling	[57]
Dapagliflozin	Rats	↓Intrarenal RAS & oxidative markers	[39]
Empagliflozin	Rats	↑SOD2 and catalase	[28]
Phlorizin	Rats	↑SODs, catalase and GSH peroxidase	[29]
Empagliflozin	Mice	↓Insulin resistance via inducing fat browning	[66,69]
Dapagliflozin	Mice	Promoting β-cell regeneration & α-to-β cell conversion	[73]
Empagliflozin	Mice	↓Oxidative stress from glucotoxicity & preserving β-cell	[72]
Empagliflozin	T2DM patients	Improving GSH & GSH reductase profiles in leukocytes	[62]
Empagliflozin	T2DM patients	↓Insulin resistance in skeletal muscle cells	[64]
Empagliflozin	T2DM patients	↓Insulin resistance & ↑β-cell sensitivity	[65]
Canagliflozin	T2DM patients	↓Insulin resistance & ↓visceral fat mass	[67]
Dapagliflozin	T2DM patients	↓Urinary 8-OHdG & improvement of endothelial function	[75]
Canagliflozin	T2DM patients	↓ox-LDL & improvement of renal/cardiac biomarkers	[76]

8-OHdG, 8-hydroxy-2-deoxyguanosine; AGE, advanced glycation end product; AKT, protein kinase B; AMPK, adenosine monophosphate-activated protein kinase; eNOS, endothelial nitric oxide synthase; GSH, glutathione; HO-1, heme oxygenase-1; mTOR, mammalian target of rapamycin; NOX4, nicotinamide adenine dinucleotide phosphate oxidase 4; Nrf2, nuclear factor erythroid 2-related factor 2; ox-LDL, oxidized low-density lipoprotein; PI3K, phosphatidylinositol 3 kinase; RAGE, receptor for advanced glycation end product; SGLT2 inhibitor, sodium-glucose cotransporter 2 inhibitors; SOD, superoxide dismutase; T2DM, type 2 diabetes mellitus.

**Table 2:** Preclinical studies demonstrating SGLT2 inhibitors as antioxidants in nonalcoholic fatty liver disease (NAFLD).

SGLT2 Inhibitor	Species	Findings	References
Dapagliflozin	Mice	Reduce MPO and ROS production	[160]
Ipragliflozin	Rats	Reduce TBARS and PCG	[26]
Remogliflozin	Mice	Reduce TBARS and ORAC	[161]
Canagliflozin	Rats	Reduce hepatic MDA while increase SOD and GPx activities	[162]
Canagliflozin	Mice	Inhibit complex I to activate AMPK/ACC pathways	[163]
NGI001	Human	Inhibit complex I *(?) to activate AMPK/ACC pathways	[164]

ACC, acetyl-CoA carboxylase; AMPK, adenosine monophosphate-activated protein kinase; GPx, glutathione peroxidase; MDA, malondialdehyde; MPO, myeloperoxidase; ORAC, oxygen radical absorbance capacity; PCG, protein carbonyl groups; ROS, reactive oxygen species; SOD, superoxide dismutase; TBARS, thiobarbituric acid-reactive substances; *(?) indicates the probable inhibition of complex I by NGI001 was not examined in the study.

**Table 3:** Preclinical evidence for the therapeutic implications of SGLT2 inhibitors in neural diseases.

Disease	Study Subject	SGLT2 Inhibitor	Findings	References
CI	mice	empagliflozin	Reduce cerebral oxidative stress and increase cerebral BDNF	[174,175]
Stroke	Rats	empagliflozin	Upregulate HIF-1α and VEGF; decreased MDA, increased activity of catalase and elevated GSH	[178,179]
AD	Mice Rats	empagliflozin canaglifozin	Reduce senile plaque density and amyloid β levels; inhibit acetylcholinesterase	[175] [182]
PD	Rats	dapagliflozin	Reduce ROS-dependent neuronal apoptosis and upregulate GDNF/PI3K/AKT/GSK-3β pathway	[183]
Epilepsy	Rats	dapagliflozin	Reduce glucose and sodium transported into the neurons.	[184]

AD, Alzheimer’s disease; BDNF, brain-derived neurotrophic factor; CI, cognitive impairment; GDNF, glial cell-derived neurotrophic factor; HIF-1α, hypoxia-inducible factor-1α; MDA, malondialdehyde; PD, Parkinson’s disease; VEGF, vascular endothelial growth factor.

## References

[1] Cowie M.R., Fisher M. (2020). Sglt2 inhibitors: Mechanisms of cardiovascular benefit beyond glycaemic control. Nat. Rev. Cardiol..

[2] Alicic R.Z., Johnson E.J., Tuttle K.R. (2018). Sglt2 inhibition for the prevention and treatment of diabetic kidney disease: A review. Am. J. Kidney Dis..

[3] Bailey C.J. (2019). Uric acid and the cardio-renal effects of sglt2 inhibitors. Diabetes Obes. Metab..

[4] Thomas M.C., Cherney D.Z.I. (2018). The actions of sglt2 inhibitors on metabolism, renal function and blood pressure. Diabetologia.

[5] Saisho Y. (2020). Sglt2 inhibitors: The star in the treatment of type 2 diabetes?. Diseases.

[6] Cannon C.P., McGuire D.K., Pratley R., Dagogo-Jack S., Mancuso J., Huyck S., Charbonnel B., Shih W.J., Gallo S., Masiukiewicz U. (2018). Design and baseline characteristics of the evaluation of ertugliflozin efficacy and safety cardiovascular outcomes trial (vertis-cv). Am. Heart J..

[7] McMurray J.J.V., Solomon S.D., Inzucchi S.E., Kober L., Kosiborod M.N., Martinez F.A., Ponikowski P., Sabatine M.S., Anand I.S., Belohlavek J. (2019). Dapagliflozin in patients with heart failure and reduced ejection fraction. N. Engl. J. Med..

[8] Neal B., Perkovic V., Mahaffey K.W., de Zeeuw D., Fulcher G., Erondu N., Shaw W., Law G., Desai M., Matthews D.R. (2017). Canagliflozin and cardiovascular and renal events in type 2 diabetes. N. Engl. J. Med..

[9] Perkovic V., de Zeeuw D., Mahaffey K.W., Fulcher G., Erondu N., Shaw W., Barrett T.D., Weidner-Wells M., Deng H., Matthews D.R. (2018). Canagliflozin and renal outcomes in type 2 diabetes: Results from the canvas program randomised clinical trials. Lancet Diabetes Endocrinol..

[10] Wiviott S.D., Raz I., Bonaca M.P., Mosenzon O., Kato E.T., Cahn A., Silverman M.G., Zelniker T.A., Kuder J.F., Murphy S.A. (2019). Dapagliflozin and cardiovascular outcomes in type 2 diabetes. N. Engl. J. Med..

[11] Yu Y., Tse K.Y., Lee H.H.Y., Chow K.L., Tsang H.W., Wong R.W.C., Cheung E.T.Y., Cheuk W., Lee V.W.K., Chan W.K. (2020). Predictive biomarkers and tumor microenvironment in female genital melanomas: A multi-institutional study of 55 cases. Mod. Pathol..

[12] Zinman B., Wanner C., Lachin J.M., Fitchett D., Bluhmki E., Hantel S., Mattheus M., Devins T., Johansen O.E., Woerle H.J. (2015). Empagliflozin, cardiovascular outcomes, and mortality in type 2 diabetes. N. Engl. J. Med..

[13] Buse J.B., Wexler D.J., Tsapas A., Rossing P., Mingrone G., Mathieu C., D’Alessio D.A., Davies M.J. (2020). 2019 update to: Management of hyperglycaemia in type 2 diabetes, 2018. A consensus report by the american diabetes association (ada) and the european association for the study of diabetes (easd). Diabetologia.

[14] Lee W.C., Chau Y.Y., Ng H.Y., Chen C.H., Wang P.W., Liou C.W., Lin T.K., Chen J.B. (2019). Empagliflozin protects hk-2 cells from high glucose-mediated injuries via a mitochondrial mechanism. Cells.

[15] Liu X., Xu C., Xu L., Li X., Sun H., Xue M., Li T., Yu X., Sun B., Chen L. (2020). Empagliflozin improves diabetic renal tubular injury by alleviating mitochondrial fission via ampk/sp1/pgam5 pathway. Metabolism.

[16] Lee Y.H., Kim S.H., Kang J.M., Heo J.H., Kim D.J., Park S.H., Sung M., Kim J., Oh J., Yang D.H. (2019). Empagliflozin attenuates diabetic tubulopathy by improving mitochondrial fragmentation and autophagy. Am. J. Physiol. Renal Physiol..

[17] Margonato D., Galati G., Mazzetti S., Cannistraci R., Perseghin G., Margonato A., Mortara A. (2021). Renal protection: A leading mechanism for cardiovascular benefit in patients treated with sglt2 inhibitors. Heart Fail. Rev..

[18] Scheen A.J. (2019). Effect of sglt2 inhibitors on the sympathetic nervous system and blood pressure. Curr. Cardiol. Rep..

[19] Zelniker T.A., Braunwald E. (2020). Mechanisms of cardiorenal effects of sodium-glucose cotransporter 2 inhibitors: Jacc state-of-the-art review. J. Am. Coll. Cardiol..

[20] Verma S., McMurray J.J.V. (2018). Sglt2 inhibitors and mechanisms of cardiovascular benefit: A state-of-the-art review. Diabetologia.

[21] Li C., Zhang J., Xue M., Li X., Han F., Liu X., Xu L., Lu Y., Cheng Y., Li T. (2019). Sglt2 inhibition with empagliflozin attenuates myocardial oxidative stress and fibrosis in diabetic mice heart. Cardiovasc. Diabetol..

[22] Ishibashi Y., Matsui T., Yamagishi S. (2016). Tofogliflozin, a highly selective inhibitor of sglt2 blocks proinflammatory and proapoptotic effects of glucose overload on proximal tubular cells partly by suppressing oxidative stress generation. Horm. Metab. Res..

[23] Sies H. (2015). Oxidative stress: A concept in redox biology and medicine. Redox Biol..

[24] Steven S., Oelze M., Hanf A., Kröller-Schön S., Kashani F., Roohani S., Welschof P., Kopp M., Gödtel-Armbrust U., Xia N. (2017). The sglt2 inhibitor empagliflozin improves the primary diabetic complications in zdf rats. Redox Biol..

[25] Terami N., Ogawa D., Tachibana H., Hatanaka T., Wada J., Nakatsuka A., Eguchi J., Horiguchi C.S., Nishii N., Yamada H. (2014). Long-term treatment with the sodium glucose cotransporter 2 inhibitor, dapagliflozin, ameliorates glucose homeostasis and diabetic nephropathy in db/db mice. PLoS ONE.

[26] Tahara A., Kurosaki E., Yokono M., Yamajuku D., Kihara R., Hayashizaki Y., Takasu T., Imamura M., Li Q., Tomiyama H. (2014). Effects of sodium-glucose cotransporter 2 selective inhibitor ipragliflozin on hyperglycaemia, oxidative stress, inflammation and liver injury in streptozotocin-induced type 1 diabetic rats. J. Pharm. Pharmacol..

[27] Yaribeygi H., Atkin S.L., Butler A.E., Sahebkar A. (2019). Sodium-glucose cotransporter inhibitors and oxidative stress: An update. J. Cell Physiol..

[28] Oshima H., Miki T., Kuno A., Mizuno M., Sato T., Tanno M., Yano T., Nakata K., Kimura Y., Abe K. (2019). Empagliflozin, an sglt2 inhibitor, reduced the mortality rate after acute myocardial infarction with modification of cardiac metabolomes and antioxidants in diabetic rats. J. Pharmacol. Exp. Ther..

[29] Osorio H., Coronel I., Arellano A., Pacheco U., Bautista R., Franco M., Escalante B. (2012). Sodium-glucose cotransporter inhibition prevents oxidative stress in the kidney of diabetic rats. Oxid. Med. Cell. Longev..

[30] Scafoglio C.R., Villegas B., Abdelhady G., Bailey S.T., Liu J., Shirali A.S., Wallace W.D., Magyar C.E., Grogan T.R., Elashoff D. (2018). Sodium-glucose transporter 2 is a diagnostic and therapeutic target for early-stage lung adenocarcinoma. Sci. Transl. Med..

[31] Koepsell H. (2017). The na(+)-d-glucose cotransporters sglt1 and sglt2 are targets for the treatment of diabetes and cancer. Pharmacol. Ther..

[32] Komatsu S., Nomiyama T., Numata T., Kawanami T., Hamaguchi Y., Iwaya C., Horikawa T., Fujimura-Tanaka Y., Hamanoue N., Motonaga R. (2020). Sglt2 inhibitor ipragliflozin attenuates breast cancer cell proliferation. Endocr. J..

[33] Kaji K., Nishimura N., Seki K., Sato S., Saikawa S., Nakanishi K., Furukawa M., Kawaratani H., Kitade M., Moriya K. (2018). Sodium glucose cotransporter 2 inhibitor canagliflozin attenuates liver cancer cell growth and angiogenic activity by inhibiting glucose uptake. Int. J. Cancer.

[34] Villani L.A., Smith B.K., Marcinko K., Ford R.J., Broadfield L.A., Green A.E., Houde V.P., Muti P., Tsakiridis T., Steinberg G.R. (2016). The diabetes medication canagliflozin reduces cancer cell proliferation by inhibiting mitochondrial complex-i supported respiration. Mol. Metab..

[35] Ojima A., Matsui T., Nishino Y., Nakamura N., Yamagishi S. (2015). Empagliflozin, an inhibitor of sodium-glucose cotransporter 2 exerts anti-inflammatory and antifibrotic effects on experimental diabetic nephropathy partly by suppressing ages-receptor axis. Horm. Metab. Res..

[36] Han J.H., Oh T.J., Lee G., Maeng H.J., Lee D.H., Kim K.M., Choi S.H., Jang H.C., Lee H.S., Park K.S. (2017). The beneficial effects of empagliflozin, an sglt2 inhibitor, on atherosclerosis in apoe (-/-) mice fed a western diet. Diabetologia.

[37] Vallon V., Rose M., Gerasimova M., Satriano J., Platt K.A., Koepsell H., Cunard R., Sharma K., Thomson S.C., Rieg T. (2013). Knockout of na-glucose transporter sglt2 attenuates hyperglycemia and glomerular hyperfiltration but not kidney growth or injury in diabetes mellitus. Am. J. Physiol. Renal Physiol..

[38] Yaribeygi H., Butler A.E., Atkin S.L., Katsiki N., Sahebkar A. (2018). Sodium-glucose cotransporter 2 inhibitors and inflammation in chronic kidney disease: Possible molecular pathways. J. Cell Physiol..

[39] Shin S.J., Chung S., Kim S.J., Lee E.M., Yoo Y.H., Kim J.W., Ahn Y.B., Kim E.S., Moon S.D., Kim M.J. (2016). Effect of sodium-glucose co-transporter 2 inhibitor, dapagliflozin, on renal renin-angiotensin system in an animal model of type 2 diabetes. PLoS ONE.

[40] Reuter S., Gupta S.C., Chaturvedi M.M., Aggarwal B.B. (2010). Oxidative stress, inflammation, and cancer: How are they linked?. Free Radic. Biol. Med..

[41] Dandekar A., Mendez R., Zhang K. (2015). Cross talk between er stress, oxidative stress, and inflammation in health and disease. Methods Mol. Biol..

[42] Singh M., Kumar A. (2018). Risks associated with sglt2 inhibitors: An overview. Curr. Drug Saf..

[43] Filippas-Ntekouan S., Filippatos T.D., Elisaf M.S. (2018). Sglt2 inhibitors: Are they safe?. Postgrad. Med..

[44] Vardeny O. (2020). The sweet spot: Heart failure prevention with sglt2 inhibitors. Am. J. Med..

[45] Lytvyn Y., Bjornstad P., Udell J.A., Lovshin J.A., Cherney D.Z.I. (2017). Sodium glucose cotransporter-2 inhibition in heart failure: Potential mechanisms, clinical applications, and summary of clinical trials. Circulation.

[46] Rastogi A., Bhansali A. (2017). Sglt2 inhibitors through the windows of empa-reg and canvas trials: A review. Diabetes Ther..

[47] Saeedi P., Petersohn I., Salpea P., Malanda B., Karuranga S., Unwin N., Colagiuri S., Guariguata L., Motala A.A., Ogurtsova K. (2019). Global and regional diabetes prevalence estimates for 2019 and projections for 2030 and 2045: Results from the international diabetes federation diabetes atlas, 9(th) edition. Diabetes Res. Clin. Pract..

[48] Rehman K., Akash M.S.H. (2017). Mechanism of generation of oxidative stress and pathophysiology of type 2 diabetes mellitus: How are they interlinked?. J. Cell Biochem..

[49] Luc K., Schramm-Luc A., Guzik T.J., Mikolajczyk T.P. (2019). Oxidative stress and inflammatory markers in prediabetes and diabetes. J. Physiol. Pharmacol..

[50] Anderson E.J., Lustig M.E., Boyle K.E., Woodlief T.L., Kane D.A., Lin C.T., Price J.W., Kang L., Rabinovitch P.S., Szeto H.H. (2009). Mitochondrial h2o2 emission and cellular redox state link excess fat intake to insulin resistance in both rodents and humans. J. Clin. Investig..

[51] Jansen F., Yang X., Franklin B.S., Hoelscher M., Schmitz T., Bedorf J., Nickenig G., Werner N. (2013). High glucose condition increases nadph oxidase activity in endothelial microparticles that promote vascular inflammation. Cardiovasc. Res..

[52] Wan A., Rodrigues B. (2016). Endothelial cell-cardiomyocyte crosstalk in diabetic cardiomyopathy. Cardiovasc. Res..

[53] Wenclewska S., Szymczak-Pajor I., Drzewoski J., Bunk M., Śliwińska A. (2019). Vitamin d supplementation reduces both oxidative DNA damage and insulin resistance in the elderly with metabolic disorders. Int. J. Mol. Sci..

[54] He L., Zhang G., Wei M., Zhao Y., Chen W., Peng Q., Meng G. (2019). Effect of individualized dietary intervention on oxidative stress in patients with type 2 diabetes complicated by tuberculosis in xinjiang, china. Diabetes Ther..

[55] Bordalo Tonucci L., Dos Santos K.M., De Luces Fortes Ferreira C.L., Ribeiro S.M., De Oliveira L.L., Martino H.S. (2017). Gut microbiota and probiotics: Focus on diabetes mellitus. Crit. Rev. Food Sci. Nutr..

[56] Sun X., Han F., Lu Q., Li X., Ren D., Zhang J., Han Y., Xiang Y.K., Li J. (2020). Empagliflozin ameliorates obesity-related cardiac dysfunction by regulating sestrin2-mediated ampk-mtor signaling and redox homeostasis in high-fat diet-induced obese mice. Diabetes.

[57] Li C.Y., Wang L.X., Dong S.S., Hong Y., Zhou X.H., Zheng W.W., Zheng C. (2018). Phlorizin exerts direct protective effects on palmitic acid (pa)-induced endothelial dysfunction by activating the pi3k/akt/enos signaling pathway and increasing the levels of nitric oxide (no). Med. Sci. Monit. Basic Res..

[58] Kawanami D., Matoba K., Takeda Y., Nagai Y., Akamine T., Yokota T., Sango K., Utsunomiya K. (2017). Sglt2 inhibitors as a therapeutic option for diabetic nephropathy. Int. J. Mol. Sci..

[59] Shao Q., Meng L., Lee S., Tse G., Gong M., Zhang Z., Zhao J., Zhao Y., Li G., Liu T. (2019). Empagliflozin, a sodium glucose co-transporter-2 inhibitor, alleviates atrial remodeling and improves mitochondrial function in high-fat diet/streptozotocin-induced diabetic rats. Cardiovasc. Diabetol..

[60] Sa-Nguanmoo P., Tanajak P., Kerdphoo S., Jaiwongkam T., Pratchayasakul W., Chattipakorn N., Chattipakorn S.C. (2017). Sglt2-inhibitor and dpp-4 inhibitor improve brain function via attenuating mitochondrial dysfunction, insulin resistance, inflammation, and apoptosis in hfd-induced obese rats. Toxicol. Appl. Pharmacol..

[61] Tapia E., Sánchez-Lozada L.G., García-Niño W.R., García E., Cerecedo A., García-Arroyo F.E., Osorio H., Arellano A., Cristóbal-García M., Loredo M.L. (2014). Curcumin prevents maleate-induced nephrotoxicity: Relation to hemodynamic alterations, oxidative stress, mitochondrial oxygen consumption and activity of respiratory complex i. Free Radic. Res..

[62] Iannantuoni F., A M.d.M., Diaz-Morales N., Falcon R., Bañuls C., Abad-Jimenez Z., Victor V.M., Hernandez-Mijares A., Rovira-Llopis S. (2019). The sglt2 inhibitor empagliflozin ameliorates the inflammatory profile in type 2 diabetic patients and promotes an antioxidant response in leukocytes. J. Clin. Med..

[63] Yaribeygi H., Sathyapalan T., Maleki M., Jamialahmadi T., Sahebkar A. (2020). Molecular mechanisms by which sglt2 inhibitors can induce insulin sensitivity in diabetic milieu: A mechanistic review. Life Sci..

[64] Goto Y., Otsuka Y., Ashida K., Nagayama A., Hasuzawa N., Iwata S., Hara K., Tsuruta M., Wada N., Motomura S. (2020). Improvement of skeletal muscle insulin sensitivity by 1 week of sglt2 inhibitor use. Endocr. Connect..

[65] Ferrannini E., Muscelli E., Frascerra S., Baldi S., Mari A., Heise T., Broedl U.C., Woerle H.J. (2014). Metabolic response to sodium-glucose cotransporter 2 inhibition in type 2 diabetic patients. J. Clin. Investig..

[66] Kern M., Klöting N., Mark M., Mayoux E., Klein T., Blüher M. (2016). The sglt2 inhibitor empagliflozin improves insulin sensitivity in db/db mice both as monotherapy and in combination with linagliptin. Metabolism.

[67] Koike Y., Shirabe S.I., Maeda H., Yoshimoto A., Arai K., Kumakura A., Hirao K., Terauchi Y. (2019). Effect of canagliflozin on the overall clinical state including insulin resistance in japanese patients with type 2 diabetes mellitus. Diabetes Res. Clin. Pract..

[68] Singh A.K., Unnikrishnan A.G., Zargar A.H., Kumar A., Das A.K., Saboo B., Sinha B., Gangopadhyay K.K., Talwalkar P.G., Ghosal S. (2019). Evidence-based consensus on positioning of sglt2i in type 2 diabetes mellitus in indians. Diabetes Ther..

[69] Xu L., Ota T. (2018). Emerging roles of sglt2 inhibitors in obesity and insulin resistance: Focus on fat browning and macrophage polarization. Adipocyte.

[70] Al Jobori H., Daniele G., Adams J., Cersosimo E., Solis-Herrera C., Triplitt C., DeFronzo R.A., Abdul-Ghani M. (2018). Empagliflozin treatment is associated with improved β-cell function in type 2 diabetes mellitus. J. Clin. Endocrinol. Metab..

[71] Takahara M., Shiraiwa T., Matsuoka T.A., Katakami N., Shimomura I. (2015). Ameliorated pancreatic β cell dysfunction in type 2 diabetic patients treated with a sodium-glucose cotransporter 2 inhibitor ipragliflozin. Endocr. J..

[72] Cheng S.T., Chen L., Li S.Y., Mayoux E., Leung P.S. (2016). The effects of empagliflozin, an sglt2 inhibitor, on pancreatic β-cell mass and glucose homeostasis in type 1 diabetes. PLoS ONE.

[73] Wei R., Cui X., Feng J., Gu L., Lang S., Wei T., Yang J., Liu J., Le Y., Wang H. (2020). Dapagliflozin promotes beta cell regeneration by inducing pancreatic endocrine cell phenotype conversion in type 2 diabetic mice. Metabolism.

[74] de Boer I.H., Caramori M.L., Chan J.C., Heerspink H.J., Hurst C., Khunti K., Liew A., Michos E.D., Navaneethan S.D., Olowu W.A. (2020). Kdigo 2020 clinical practice guideline for diabetes management in chronic kidney disease. Kidney Int..

[75] Shigiyama F., Kumashiro N., Miyagi M., Ikehara K., Kanda E., Uchino H., Hirose T. (2017). Effectiveness of dapagliflozin on vascular endothelial function and glycemic control in patients with early-stage type 2 diabetes mellitus: Defence study. Cardiovasc. Diabetol..

[76] Sezai A., Sekino H., Unosawa S., Taoka M., Osaka S., Tanaka M. (2019). Canagliflozin for japanese patients with chronic heart failure and type ii diabetes. Cardiovasc. Diabetol..

[77] Evans M., Hicks D., Patel D., Patel V., McEwan P., Dashora U. (2020). Optimising the benefits of sglt2 inhibitors for type 1 diabetes. Diabetes Ther..

[78] Taylor S.I., Blau J.E., Rother K.I., Beitelshees A.L. (2019). Sglt2 inhibitors as adjunctive therapy for type 1 diabetes: Balancing benefits and risks. Lancet Diabetes Endocrinol..

[79] Marshall S.M., Flyvbjerg A. (2006). Prevention and early detection of vascular complications of diabetes. BMJ.

[80] Romero S.P., Andrey J.L., Garcia-Egido A., Escobar M.A., Perez V., Corzo R., Garcia-Domiguez G.J., Gomez F. (2013). Metformin therapy and prognosis of patients with heart failure and new-onset diabetes mellitus. A propensity-matched study in the community. Int. J. Cardiol..

[81] UK Prospective Diabetes Study (UKPDS) Group (1998). Uk Prospective Diabetes Study 33. Intensive blood-glucose control with sulphonylureas or insulin compared with conventional treatment and risk of complications in patients with type 2 diabetes. Lancet.

[82] Phung O.J., Schwartzman E., Allen R.W., Engel S.S., Rajpathak S.N. (2013). Sulphonylureas and risk of cardiovascular disease: Systematic review and meta-analysis. Diabet. Med..

[83] Home P.D., Pocock S.J., Beck-Nielsen H., Gomis R., Hanefeld M., Jones N.P., Komajda M., McMurray J.J., Group R.S. (2007). Rosiglitazone evaluated for cardiovascular outcomes--an interim analysis. N. Engl. J. Med..

[84] Mahaffey K.W., Hafley G., Dickerson S., Burns S., Tourt-Uhlig S., White J., Newby L.K., Komajda M., McMurray J., Bigelow R. (2013). Results of a reevaluation of cardiovascular outcomes in the record trial. Am. Heart J..

[85] Green J.B., Bethel M.A., Armstrong P.W., Buse J.B., Engel S.S., Garg J., Josse R., Kaufman K.D., Koglin J., Korn S. (2015). Effect of sitagliptin on cardiovascular outcomes in type 2 diabetes. N. Engl. J. Med..

[86] Scirica B.M., Braunwald E., Raz I., Cavender M.A., Morrow D.A., Jarolim P., Udell J.A., Mosenzon O., Im K., Umez-Eronini A.A. (2014). Heart failure, saxagliptin, and diabetes mellitus: Observations from the savor-timi 53 randomized trial. Circulation.

[87] Akinci B. (2019). Dapagliflozin and cardiovascular outcomes in type 2 diabetes. Reply. N. Engl. J. Med..

[88] Packer M., Anker S.D., Butler J., Filippatos G., Pocock S.J., Carson P., Januzzi J., Verma S., Tsutsui H., Brueckmann M. (2020). Cardiovascular and renal outcomes with empagliflozin in heart failure. N. Engl. J. Med..

[89] Frati G., Schirone L., Chimenti I., Yee D., Biondi-Zoccai G., Volpe M., Sciarretta S. (2017). An overview of the inflammatory signalling mechanisms in the myocardium underlying the development of diabetic cardiomyopathy. Cardiovasc. Res..

[90] Dey S., DeMazumder D., Sidor A., Foster D.B., O’Rourke B. (2018). Mitochondrial ros drive sudden cardiac death and chronic proteome remodeling in heart failure. Circ. Res..

[91] Shah M.S., Brownlee M. (2016). Molecular and cellular mechanisms of cardiovascular disorders in diabetes. Circ. Res..

[92] Paulus W.J., Tschope C. (2013). A novel paradigm for heart failure with preserved ejection fraction: Comorbidities drive myocardial dysfunction and remodeling through coronary microvascular endothelial inflammation. J. Am. Coll. Cardiol..

[93] Joshi M., Kotha S.R., Malireddy S., Selvaraju V., Satoskar A.R., Palesty A., McFadden D.W., Parinandi N.L., Maulik N. (2014). Conundrum of pathogenesis of diabetic cardiomyopathy: Role of vascular endothelial dysfunction, reactive oxygen species, and mitochondria. Mol. Cell Biochem..

[94] Munzel T., Gori T., Keaney J.F., Maack C., Daiber A. (2015). Pathophysiological role of oxidative stress in systolic and diastolic heart failure and its therapeutic implications. Eur. Heart J..

[95] Belch J.J., Bridges A.B., Scott N., Chopra M. (1991). Oxygen free radicals and congestive heart failure. Br. Heart J..

[96] van der Pol A., van Gilst W.H., Voors A.A., van der Meer P. (2019). Treating oxidative stress in heart failure: Past, present and future. Eur. J. Heart Fail..

[97] Packer M. (2020). Sglt2 inhibitors produce cardiorenal benefits by promoting adaptive cellular reprogramming to induce a state of fasting mimicry: A paradigm shift in understanding their mechanism of action. Diabetes Care.

[98] Alcendor R.R., Gao S., Zhai P., Zablocki D., Holle E., Yu X., Tian B., Wagner T., Vatner S.F., Sadoshima J. (2007). Sirt1 regulates aging and resistance to oxidative stress in the heart. Circ. Res..

[99] Meijles D.N., Zoumpoulidou G., Markou T., Rostron K.A., Patel R., Lay K., Handa B.S., Wong B., Sugden P.H., Clerk A. (2019). The cardiomyocyte “redox rheostat”: Redox signalling via the ampk-mtor axis and regulation of gene and protein expression balancing survival and death. J. Mol. Cell Cardiol..

[100] Tian R., Musi N., D’Agostino J., Hirshman M.F., Goodyear L.J. (2001). Increased adenosine monophosphate-activated protein kinase activity in rat hearts with pressure-overload hypertrophy. Circulation.

[101] Beauloye C., Bertrand L., Horman S., Hue L. (2011). Ampk activation, a preventive therapeutic target in the transition from cardiac injury to heart failure. Cardiovasc. Res..

[102] Gelinas R., Mailleux F., Dontaine J., Bultot L., Demeulder B., Ginion A., Daskalopoulos E.P., Esfahani H., Dubois-Deruy E., Lauzier B. (2018). Ampk activation counteracts cardiac hypertrophy by reducing o-glcnacylation. Nat. Commun..

[103] Cordero M.D., Williams M.R., Ryffel B. (2018). Amp-activated protein kinase regulation of the nlrp3 inflammasome during aging. Trends Endocrinol. Metab..

[104] He C., Li H., Viollet B., Zou M.H., Xie Z. (2015). Ampk suppresses vascular inflammation in vivo by inhibiting signal transducer and activator of transcription-1. Diabetes.

[105] Vlassara H., Bucala R. (1996). Recent progress in advanced glycation and diabetic vascular disease: Role of advanced glycation end product receptors. Diabetes.

[106] Yan S.F., Ramasamy R., Naka Y., Schmidt A.M. (2003). Glycation, inflammation, and rage: A scaffold for the macrovascular complications of diabetes and beyond. Circ. Res..

[107] Yamagishi S., Nakamura K., Matsui T., Noda Y., Imaizumi T. (2008). Receptor for advanced glycation end products (rage): A novel therapeutic target for diabetic vascular complication. Curr. Pharm. Des..

[108] Goldin A., Beckman J.A., Schmidt A.M., Creager M.A. (2006). Advanced glycation end products: Sparking the development of diabetic vascular injury. Circulation.

[109] Paradela-Dobarro B., Agra R.M., Alvarez L., Varela-Roman A., Garcia-Acuna J.M., Gonzalez-Juanatey J.R., Alvarez E., Garcia-Seara F.J. (2019). The different roles for the advanced glycation end products axis in heart failure and acute coronary syndrome settings. Nutr. Metab. Cardiovasc. Dis..

[110] Brito D., Bettencourt P., Carvalho D., Ferreira J., Fontes-Carvalho R., Franco F., Moura B., Silva-Cardoso J.C., de Melo R.T., Fonseca C. (2020). Sodium-glucose co-transporter 2 inhibitors in the failing heart: A growing potential. Cardiovasc. Drugs Ther..

[111] Aragon-Herrera A., Feijoo-Bandin S., Otero Santiago M., Barral L., Campos-Toimil M., Gil-Longo J., Costa Pereira T.M., Garcia-Caballero T., Rodriguez-Segade S., Rodriguez J. (2019). Empagliflozin reduces the levels of cd36 and cardiotoxic lipids while improving autophagy in the hearts of zucker diabetic fatty rats. Biochem. Pharmacol..

[112] Inoue M.K., Matsunaga Y., Nakatsu Y., Yamamotoya T., Ueda K., Kushiyama A., Sakoda H., Fujishiro M., Ono H., Iwashita M. (2019). Possible involvement of normalized pin1 expression level and ampk activation in the molecular mechanisms underlying renal protective effects of sglt2 inhibitors in mice. Diabetol. Metab. Syndr..

[113] Mizuno M., Kuno A., Yano T., Miki T., Oshima H., Sato T., Nakata K., Kimura Y., Tanno M., Miura T. (2018). Empagliflozin normalizes the size and number of mitochondria and prevents reduction in mitochondrial size after myocardial infarction in diabetic hearts. Physiol. Rep..

[114] Zhou H., Wang S., Zhu P., Hu S., Chen Y., Ren J. (2018). Empagliflozin rescues diabetic myocardial microvascular injury via ampk-mediated inhibition of mitochondrial fission. Redox Biol..

[115] Packer M. (2017). Activation and inhibition of sodium-hydrogen exchanger is a mechanism that links the pathophysiology and treatment of diabetes mellitus with that of heart failure. Circulation.

[116] Gui L., Liu B., Lv G. (2016). Hypoxia induces autophagy in cardiomyocytes via a hypoxia-inducible factor 1-dependent mechanism. Exp. Ther. Med..

[117] Salim H.M., Fukuda D., Yagi S., Soeki T., Shimabukuro M., Sata M. (2016). Glycemic control with ipragliflozin, a novel selective sglt2 inhibitor, ameliorated endothelial dysfunction in streptozotocin-induced diabetic mouse. Front. Cardiovasc. Med..

[118] Habibi J., Aroor A.R., Sowers J.R., Jia G., Hayden M.R., Garro M., Barron B., Mayoux E., Rector R.S., Whaley-Connell A. (2017). Sodium glucose transporter 2 (sglt2) inhibition with empagliflozin improves cardiac diastolic function in a female rodent model of diabetes. Cardiovasc. Diabetol..

[119] Andreadou I., Efentakis P., Balafas E., Togliatto G., Davos C.H., Varela A., Dimitriou C.A., Nikolaou P.E., Maratou E., Lambadiari V. (2017). Empagliflozin limits myocardial infarction in vivo and cell death in vitro: Role of stat3, mitochondria, and redox aspects. Front. Physiol..

[120] Nojima T., Matsubayashi Y., Yoshida A., Suganami H., Abe T., Ishizawa M., Fujihara K., Tanaka S., Kaku K., Sone H. (2020). Influence of an sglt2 inhibitor, tofogliflozin, on the resting heart rate in relation to adipose tissue insulin resistance. Diabet. Med..

[121] Falcao-Pires I., Leite-Moreira A.F. (2012). Diabetic cardiomyopathy: Understanding the molecular and cellular basis to progress in diagnosis and treatment. Heart Fail. Rev..

[122] O’Meara E., McDonald M., Chan M., Ducharme A., Ezekowitz J.A., Giannetti N., Grzeslo A., Heckman G.A., Howlett J.G., Koshman S.L. (2020). Ccs/chfs heart failure guidelines: Clinical trial update on functional mitral regurgitation, sglt2 inhibitors, arni in hfpef, and tafamidis in amyloidosis. Can. J. Cardiol..

[123] Santos-Gallego C.G., Garcia-Ropero A., Mancini D., Pinney S.P., Contreras J.P., Fergus I., Abascal V., Moreno P., Atallah-Lajam F., Tamler R. (2019). Rationale and design of the empa-tropism trial (atru-4): Are the “cardiac benefits” of empagliflozin independent of its hypoglycemic activity?. Cardiovasc. Drugs Ther..

[124] Osonoi T., Gouda M., Kubo M., Arakawa K., Hashimoto T., Abe M. (2018). Effect of canagliflozin on urinary albumin excretion in japanese patients with type 2 diabetes mellitus and microalbuminuria: A pilot study. Diabetes Technol. Ther..

[125] Solini A., Giannini L., Seghieri M., Vitolo E., Taddei S., Ghiadoni L., Bruno R.M. (2017). Dapagliflozin acutely improves endothelial dysfunction, reduces aortic stiffness and renal resistive index in type 2 diabetic patients: A pilot study. Cardiovasc. Diabetol..

[126] Tanaka S., Sugiura Y., Saito H., Sugahara M., Higashijima Y., Yamaguchi J., Inagi R., Suematsu M., Nangaku M., Tanaka T. (2018). Sodium-glucose cotransporter 2 inhibition normalizes glucose metabolism and suppresses oxidative stress in the kidneys of diabetic mice. Kidney Int..

[127] Maeda S., Matsui T., Takeuchi M., Yamagishi S. (2013). Sodium-glucose cotransporter 2-mediated oxidative stress augments advanced glycation end products-induced tubular cell apoptosis. Diabetes/Metab. Res. Rev..

[128] Mulder S., Hammarstedt A., Nagaraj S.B., Nair V., Ju W., Hedberg J., Greasley P.J., Eriksson J.W., Oscarsson J., Heerspink H.J.L. (2020). A metabolomics-based molecular pathway analysis of how the sodium-glucose co-transporter-2 inhibitor dapagliflozin may slow kidney function decline in patients with diabetes. Diabetes Obes. Metab..

[129] Takagi S., Li J., Takagaki Y., Kitada M., Nitta K., Takasu T., Kanasaki K., Koya D. (2018). Ipragliflozin improves mitochondrial abnormalities in renal tubules induced by a high-fat diet. J. Diabetes Investig..

[130] Packer M. (2020). Role of deranged energy deprivation signaling in the pathogenesis of cardiac and renal disease in states of perceived nutrient overabundance. Circulation.

[131] Hasan R., Lasker S., Hasan A., Zerin F., Zamila M., Parvez F., Rahman M.M., Khan F., Subhan N., Alam M.A. (2020). Canagliflozin ameliorates renal oxidative stress and inflammation by stimulating ampk-akt-enos pathway in the isoprenaline-induced oxidative stress model. Sci. Rep..

[132] Sugizaki T., Zhu S., Guo G., Matsumoto A., Zhao J., Endo M., Horiguchi H., Morinaga J., Tian Z., Kadomatsu T. (2017). Treatment of diabetic mice with the sglt2 inhibitor ta-1887 antagonizes diabetic cachexia and decreases mortality. NPJ Aging Mech. Dis..

[133] Maki T., Maeno S., Maeda Y., Yamato M., Sonoda N., Ogawa Y., Wakisaka M., Inoguchi T. (2019). Amelioration of diabetic nephropathy by sglt2 inhibitors independent of its glucose-lowering effect: A possible role of sglt2 in mesangial cells. Sci. Rep..

[134] Berardo C., Di Pasqua L.G., Cagna M., Richelmi P., Vairetti M., Ferrigno A. (2020). Nonalcoholic fatty liver disease and non-alcoholic steatohepatitis: Current issues and future perspectives in preclinical and clinical research. Int. J. Mol. Sci..

[135] Paradies G., Paradies V., Ruggiero F.M., Petrosillo G. (2014). Oxidative stress, cardiolipin and mitochondrial dysfunction in nonalcoholic fatty liver disease. World J. Gastroenterol..

[136] Wu P.J., Chen J.B., Lee W.C., Ng H.Y., Lien S.C., Tsai P.Y., Wu C.H., Lee C.T., Chiou T.T. (2018). Oxidative stress and nonalcoholic fatty liver disease in hemodialysis patients. Biomed. Res. Int..

[137] Vairetti M., Di Pasqua L.G., Cagna M., Richelmi P., Ferrigno A., Berardo C. (2021). Changes in glutathione content in liver diseases: An update. Antioxidants.

[138] Bica C., Sandu C., Suceveanu A.I., Sarbu E., Stoica R.A., Gherghiceanu F., Bohiltea R.E., Stefan S.D., Stoian A.P. (2020). Non-alcoholic fatty liver disease: A major challenge in type 2 diabetes mellitus (review). Exp. Ther. Med..

[139] Ito D., Shimizu S., Inoue K., Saito D., Yanagisawa M., Inukai K., Akiyama Y., Morimoto Y., Noda M., Shimada A. (2017). Comparison of ipragliflozin and pioglitazone effects on nonalcoholic fatty liver disease in patients with type 2 diabetes: A randomized, 24-week, open-label, active-controlled trial. Diabetes Care.

[140] Armstrong M.J., Gaunt P., Aithal G.P., Barton D., Hull D., Parker R., Hazlehurst J.M., Guo K., Abouda G., Aldersley M.A. (2016). Liraglutide safety and efficacy in patients with non-alcoholic steatohepatitis (lean): A multicentre, double-blind, randomised, placebo-controlled phase 2 study. Lancet.

[141] Scheen A.J. (2019). Beneficial effects of sglt2 inhibitors on fatty liver in type 2 diabetes: A common comorbidity associated with severe complications. Diabetes Metab..

[142] Shao S.C., Kuo L.T., Chien R.N., Hung M.J., Lai E.C. (2020). Sglt2 inhibitors in patients with type 2 diabetes with non-alcoholic fatty liver diseases: An umbrella review of systematic reviews. BMJ Open Diabetes Res. Care.

[143] Sattar N., Fitchett D., Hantel S., George J.T., Zinman B. (2018). Empagliflozin is associated with improvements in liver enzymes potentially consistent with reductions in liver fat: Results from randomised trials including the empa-reg outcome® trial. Diabetologia.

[144] Shao S.C., Chang K.C., Chien R.N., Lin S.J., Hung M.J., Chan Y.Y., Kao Yang Y.H., Lai E.C. (2020). Effects of sodium-glucose co-transporter-2 inhibitors on serum alanine aminotransferase levels in people with type 2 diabetes: A multi-institutional cohort study. Diabetes Obes. Metab..

[145] Li B., Wang Y., Ye Z., Yang H., Cui X., Wang Z., Liu L. (2018). Effects of canagliflozin on fatty liver indexes in patients with type 2 diabetes: A meta-analysis of randomized controlled trials. J. Pharm. Pharm. Sci..

[146] Itani T., Ishihara T. (2018). Efficacy of canagliflozin against nonalcoholic fatty liver disease: A prospective cohort study. Obes. Sci. Pract..

[147] Shinozaki S., Tahara T., Lefor A.K., Ogura M. (2020). Long-term empagliflozin therapy improves levels of hepatic fibrosis marker in patients with non-alcoholic fatty liver disease complicated by type 2 diabetes mellitus. J. Med. Investig..

[148] Gallo S., Calle R.A., Terra S.G., Pong A., Tarasenko L., Raji A. (2020). Effects of ertugliflozin on liver enzymes in patients with type 2 diabetes: A post-hoc pooled analysis of phase 3 trials. Diabetes Ther..

[149] Seino H. (2021). Efficacy and safety of luseogliflozin in patients with type 2 diabetes complicated by hepatic dysfunction: A single-site, single-arm, open-label, exploratory trial. Diabetes Ther..

[150] Sumida Y., Murotani K., Saito M., Tamasawa A., Osonoi Y., Yoneda M., Osonoi T. (2019). Effect of luseogliflozin on hepatic fat content in type 2 diabetes patients with non-alcoholic fatty liver disease: A prospective, single-arm trial (lead trial). Hepatol. Res..

[151] Shimizu M., Suzuki K., Kato K., Jojima T., Iijima T., Murohisa T., Iijima M., Takekawa H., Usui I., Hiraishi H. (2019). Evaluation of the effects of dapagliflozin, a sodium-glucose co-transporter-2 inhibitor, on hepatic steatosis and fibrosis using transient elastography in patients with type 2 diabetes and non-alcoholic fatty liver disease. Diabetes Obes. Metab..

[152] Arai T., Atsukawa M., Tsubota A., Mikami S., Ono H., Kawano T., Yoshida Y., Tanabe T., Okubo T., Hayama K. (2021). Effect of sodium-glucose cotransporter 2 inhibitor in patients with non-alcoholic fatty liver disease and type 2 diabetes mellitus: A propensity score-matched analysis of real-world data. Ther. Adv. Endocrinol. Metab..

[153] Taheri H., Malek M., Ismail-Beigi F., Zamani F., Sohrabi M., Reza Babaei M., Khamseh M.E. (2020). Effect of empagliflozin on liver steatosis and fibrosis in patients with non-alcoholic fatty liver disease without diabetes: A randomized, double-blind, placebo-controlled trial. Adv. Ther..

[154] Akuta N., Watanabe C., Kawamura Y., Arase Y., Saitoh S., Fujiyama S., Sezaki H., Hosaka T., Kobayashi M., Kobayashi M. (2017). Effects of a sodium-glucose cotransporter 2 inhibitor in nonalcoholic fatty liver disease complicated by diabetes mellitus: Preliminary prospective study based on serial liver biopsies. Hepatol. Commun..

[155] Akuta N., Kawamura Y., Watanabe C., Nishimura A., Okubo M., Mori Y., Fujiyama S., Sezaki H., Hosaka T., Kobayashi M. (2019). Impact of sodium glucose cotransporter 2 inhibitor on histological features and glucose metabolism of non-alcoholic fatty liver disease complicated by diabetes mellitus. Hepatol. Res..

[156] Akuta N., Kawamura Y., Fujiyama S., Sezaki H., Hosaka T., Kobayashi M., Kobayashi M., Saitoh S., Suzuki F., Suzuki Y. (2020). Sglt2 inhibitor treatment outcome in nonalcoholic fatty liver disease complicated with diabetes mellitus: The long-term effects on clinical features and liver histopathology. Intern. Med..

[157] Lizardi-Cervera J., Aguilar-Zapata D. (2009). Nonalcoholic fatty liver disease and its association with cardiovascular disease. Ann. Hepatol..

[158] Marchisello S., Di Pino A., Scicali R., Urbano F., Piro S., Purrello F., Rabuazzo A.M. (2019). Pathophysiological, molecular and therapeutic issues of nonalcoholic fatty liver disease: An overview. Int. J. Mol. Sci..

[159] Bessone F., Razori M.V., Roma M.G. (2019). Molecular pathways of nonalcoholic fatty liver disease development and progression. Cell Mol. Life Sci..

[160] Tang L., Wu Y., Tian M., Sjostrom C.D., Johansson U., Peng X.R., Smith D.M., Huang Y. (2017). Dapagliflozin slows the progression of the renal and liver fibrosis associated with type 2 diabetes. Am. J. Physiol. Endocrinol. Metab..

[161] Nakano S., Katsuno K., Isaji M., Nagasawa T., Buehrer B., Walker S., Wilkison W.O., Cheatham B. (2015). Remogliflozin etabonate improves fatty liver disease in diet-induced obese male mice. J. Clin. Exp. Hepatol..

[162] Kabil S.L., Mahmoud N.M. (2018). Canagliflozin protects against non-alcoholic steatohepatitis in type-2 diabetic rats through zinc alpha-2 glycoprotein up-regulation. Eur. J. Pharmacol..

[163] Hawley S.A., Ford R.J., Smith B.K., Gowans G.J., Mancini S.J., Pitt R.D., Day E.A., Salt I.P., Steinberg G.R., Hardie D.G. (2016). The Na_+_/glucose cotransporter inhibitor canagliflozin activates ampk by inhibiting mitochondrial function and increasing cellular amp levels. Diabetes.

[164] Chiang H., Lee J.C., Huang H.C., Huang H., Liu H.K., Huang C. (2020). Delayed intervention with a novel sglt2 inhibitor ngi001 suppresses diet-induced metabolic dysfunction and non-alcoholic fatty liver disease in mice. Br. J. Pharmacol..

[165] Siesjo B.K. (1978). Brain energy metabolism and catecholaminergic activity in hypoxia, hypercapnia and ischemia. J. Neural Transm. Suppl..

[166] Manolescu A.R., Witkowska K., Kinnaird A., Cessford T., Cheeseman C. (2007). Facilitated hexose transporters: New perspectives on form and function. Physiology.

[167] Duelli R., Kuschinsky W. (2001). Brain glucose transporters: Relationship to local energy demand. News Physiol. Sci..

[168] Wright E.M. (2001). Renal na(+)-glucose cotransporters. Am. J. Physiol. Renal Physiol..

[169] Wright E.M., Turk E. (2004). The sodium/glucose cotransport family slc5. Pflug. Arch..

[170] Yu A.S., Hirayama B.A., Timbol G., Liu J., Diez-Sampedro A., Kepe V., Satyamurthy N., Huang S.C., Wright E.M., Barrio J.R. (2013). Regional distribution of sglt activity in rat brain in vivo. Am. J. Physiol. Cell Physiol..

[171] O’Malley D., Reimann F., Simpson A.K., Gribble F.M. (2006). Sodium-coupled glucose cotransporters contribute to hypothalamic glucose sensing. Diabetes.

[172] Wicinski M., Wodkiewicz E., Gorski K., Walczak M., Malinowski B. (2020). Perspective of sglt2 inhibition in treatment of conditions connected to neuronal loss: Focus on alzheimer’s disease and ischemia-related brain injury. Pharmaceuticals.

[173] Biessels G.J., Strachan M.W., Visseren F.L., Kappelle L.J., Whitmer R.A. (2014). Dementia and cognitive decline in type 2 diabetes and prediabetic stages: Towards targeted interventions. Lancet Diabetes Endocrinol..

[174] Lin B., Koibuchi N., Hasegawa Y., Sueta D., Toyama K., Uekawa K., Ma M., Nakagawa T., Kusaka H., Kim-Mitsuyama S. (2014). Glycemic control with empagliflozin, a novel selective sglt2 inhibitor, ameliorates cardiovascular injury and cognitive dysfunction in obese and type 2 diabetic mice. Cardiovasc. Diabetol..

[175] Hierro-Bujalance C., Infante-Garcia C., Del Marco A., Herrera M., Carranza-Naval M.J., Suarez J., Alves-Martinez P., Lubian-Lopez S., Garcia-Alloza M. (2020). Empagliflozin reduces vascular damage and cognitive impairment in a mixed murine model of alzheimer’s disease and type 2 diabetes. Alzheimers Res. Ther..

[176] Katan M., Luft A. (2018). Global burden of stroke. Semin. Neurol..

[177] Libby P. (2012). Inflammation in atherosclerosis. Arterioscler. Thromb. Vasc. Biol..

[178] Abdel-Latif R.G., Rifaai R.A., Amin E.F. (2020). Empagliflozin alleviates neuronal apoptosis induced by cerebral ischemia/reperfusion injury through hif-1alpha/vegf signaling pathway. Arch. Pharm. Res..

[179] Amin E.F., Rifaai R.A., Abdel-Latif R.G. (2020). Empagliflozin attenuates transient cerebral ischemia/reperfusion injury in hyperglycemic rats via repressing oxidative-inflammatory-apoptotic pathway. Fundam. Clin. Pharmacol..

[180] Zinman B., Inzucchi S.E., Lachin J.M., Wanner C., Fitchett D., Kohler S., Mattheus M., Woerle H.J., Broedl U.C., Johansen O.E. (2017). Empagliflozin and cerebrovascular events in patients with type 2 diabetes mellitus at high cardiovascular risk. Stroke.

[181] Brookmeyer R., Johnson E., Ziegler-Graham K., Arrighi H.M. (2007). Forecasting the global burden of alzheimer’s disease. Alzheimers Dement..

[182] Arafa N.M.S., Ali E.H.A., Hassan M.K. (2017). Canagliflozin prevents scopolamine-induced memory impairment in rats: Comparison with galantamine hydrobromide action. Chem. Biol. Interact..

[183] Arab H.H., Safar M.M., Shahin N.N. (2021). Targeting ros-dependent akt/gsk-3beta/nf-kappab and dj-1/nrf2 pathways by dapagliflozin attenuates neuronal injury and motor dysfunction in rotenone-induced parkinson’s disease rat model. ACS Chem. Neurosci..

[184] Erdogan M.A., Yusuf D., Christy J., Solmaz V., Erdogan A., Taskiran E., Erbas O. (2018). Highly selective sglt2 inhibitor dapagliflozin reduces seizure activity in pentylenetetrazol-induced murine model of epilepsy. BMC Neurol..

[185] Szatrowski T.P., Nathan C.F. (1991). Production of large amounts of hydrogen peroxide by human tumor cells. Cancer Res..

[186] Ames B.N., Shigenaga M.K., Hagen T.M. (1993). Oxidants, antioxidants, and the degenerative diseases of aging. Proc. Natl. Acad. Sci. USA.

[187] Sabharwal S.S., Schumacker P.T. (2014). Mitochondrial ros in cancer: Initiators, amplifiers or an achilles’ heel?. Nat. Rev. Cancer.

[188] Shiba K., Tsuchiya K., Komiya C., Miyachi Y., Mori K., Shimazu N., Yamaguchi S., Ogasawara N., Katoh M., Itoh M. (2018). Canagliflozin, an sglt2 inhibitor, attenuates the development of hepatocellular carcinoma in a mouse model of human nash. Sci. Rep..

[189] Obara K., Shirakami Y., Maruta A., Ideta T., Miyazaki T., Kochi T., Sakai H., Tanaka T., Seishima M., Shimizu M. (2017). Preventive effects of the sodium glucose cotransporter 2 inhibitor tofogliflozin on diethylnitrosamine-induced liver tumorigenesis in obese and diabetic mice. Oncotarget.

[190] Jojima T., Wakamatsu S., Kase M., Iijima T., Maejima Y., Shimomura K., Kogai T., Tomaru T., Usui I., Aso Y. (2019). The sglt2 inhibitor canagliflozin prevents carcinogenesis in a mouse model of diabetes and non-alcoholic steatohepatitis-related hepatocarcinogenesis: Association with sglt2 expression in hepatocellular carcinoma. Int. J. Mol. Sci..

[191] Scafoglio C., Hirayama B.A., Kepe V., Liu J., Ghezzi C., Satyamurthy N., Moatamed N.A., Huang J., Koepsell H., Barrio J.R. (2015). Functional expression of sodium-glucose transporters in cancer. Proc. Natl. Acad. Sci. USA.

[192] Hsieh M.H., Choe J.H., Gadhvi J., Kim Y.J., Arguez M.A., Palmer M., Gerold H., Nowak C., Do H., Mazambani S. (2019). P63 and sox2 dictate glucose reliance and metabolic vulnerabilities in squamous cell carcinomas. Cell Rep..

[193] Kuang H., Liao L., Chen H., Kang Q., Shu X., Wang Y. (2017). Therapeutic effect of sodium glucose co-transporter 2 inhibitor dapagliflozin on renal cell carcinoma. Med. Sci. Monit..

[194] Xie Z., Wang F., Lin L., Duan S., Liu X., Li X., Li T., Xue M., Cheng Y., Ren H. (2020). An sglt2 inhibitor modulates shh expression by activating ampk to inhibit the migration and induce the apoptosis of cervical carcinoma cells. Cancer Lett..

[195] Zhou J., Zhu J., Yu S.J., Ma H.L., Chen J., Ding X.F., Chen G., Liang Y., Zhang Q. (2020). Sodium-glucose co-transporter-2 (sglt-2) inhibition reduces glucose uptake to induce breast cancer cell growth arrest through ampk/mtor pathway. Biomed. Pharmacother..

[196] Okada J., Matsumoto S., Kaira K., Saito T., Yamada E., Yokoo H., Katoh R., Kusano M., Okada S., Yamada M. (2018). Sodium glucose cotransporter 2 inhibition combined with cetuximab significantly reduced tumor size and carcinoembryonic antigen level in colon cancer metastatic to liver. Clin. Colorectal Cancer.

[197] Liou G.Y., Storz P. (2010). Reactive oxygen species in cancer. Free Radic. Res..

[198] Galadari S., Rahman A., Pallichankandy S., Thayyullathil F. (2017). Reactive oxygen species and cancer paradox: To promote or to suppress?. Free Radic. Biol. Med..

[199] Kim Y.W., Byzova T.V. (2014). Oxidative stress in angiogenesis and vascular disease. Blood.

[200] El-Kenawi A., Ruffell B. (2017). Inflammation, ros, and mutagenesis. Cancer Cell.

[201] Leng W., Wu M., Pan H., Lei X., Chen L., Wu Q., Ouyang X., Liang Z. (2019). The sglt2 inhibitor dapagliflozin attenuates the activity of ros-nlrp3 inflammasome axis in steatohepatitis with diabetes mellitus. Ann. Transl. Med..

[202] Kim S.R., Lee S.G., Kim S.H., Kim J.H., Choi E., Cho W., Rim J.H., Hwang I., Lee C.J., Lee M. (2020). Sglt2 inhibition modulates nlrp3 inflammasome activity via ketones and insulin in diabetes with cardiovascular disease. Nat. Commun..

[203] Dominguez Rieg J.A., Rieg T. (2019). What does sodium-glucose co-transporter 1 inhibition add: Prospects for dual inhibition. Diabetes Obes. Metab..

[204] Markham A., Keam S.J. (2019). Sotagliflozin: First global approval. Drugs.

[205] Weihua Z., Tsan R., Huang W.C., Wu Q., Chiu C.H., Fidler I.J., Hung M.C. (2008). Survival of cancer cells is maintained by egfr independent of its kinase activity. Cancer Cell.

[206] Lai B., Xiao Y., Pu H., Cao Q., Jing H., Liu X. (2012). Overexpression of sglt1 is correlated with tumor development and poor prognosis of ovarian carcinoma. Arch. Gynecol. Obstet..

[207] Hanabata Y., Nakajima Y., Morita K., Kayamori K., Omura K. (2012). Coexpression of sglt1 and egfr is associated with tumor differentiation in oral squamous cell carcinoma. Odontology.

[208] Liu H., Ertay A., Peng P., Li J., Liu D., Xiong H., Zou Y., Qiu H., Hancock D., Yuan X. (2019). Sglt1 is required for the survival of triple-negative breast cancer cells via potentiation of egfr activity. Mol. Oncol..

[209] Dittmann K., Mayer C., Rodemann H.P., Huber S.M. (2013). Egfr cooperates with glucose transporter sglt1 to enable chromatin remodeling in response to ionizing radiation. Radiother. Oncol..

[210] Ren J., Bollu L.R., Su F., Gao G., Xu L., Huang W.C., Hung M.C., Weihua Z. (2013). Egfr-sglt1 interaction does not respond to egfr modulators, but inhibition of sglt1 sensitizes prostate cancer cells to egfr tyrosine kinase inhibitors. Prostate.

[211] Bode D., Semmler L., Wakula P., Hegemann N., Primessnig U., Beindorff N., Powell D., Dahmen R., Ruetten H., Oeing C. (2021). Dual sglt-1 and sglt-2 inhibition improves left atrial dysfunction in hfpef. Cardiovasc. Diabetol..

